# Aged gastrocnemius muscle of mice positively responds to a late onset adapted physical training

**DOI:** 10.3389/fcell.2023.1273309

**Published:** 2023-11-13

**Authors:** Barbara Cisterna, Francesco Demetrio Lofaro, Maria Assunta Lacavalla, Federico Boschi, Manuela Malatesta, Daniela Quaglino, Carlo Zancanaro, Federica Boraldi

**Affiliations:** ^1^ Department of Neuroscience, Biomedicine and Movement Sciences, University of Verona, Verona, Italy; ^2^ Department of Life Sciences, University of Modena and Reggio Emilia, Modena, Italy; ^3^ Department of Computer Science, University of Verona, Verona, Italy

**Keywords:** ageing, skeletal muscle, physical training, matrix, proteomics, electron microscopy

## Abstract

**Introduction:** A regular physical training is known to contribute to preserve muscle mass and strength, maintaining structure and function of neural and vascular compartments and preventing muscle insulin resistance and inflammation. However, physical activity is progressively reduced during aging causing mobility limitations and poor quality of life. Although physical exercise for rehabilitation purposes (*e.g.,* after fractures or cardiovascular events) or simply aiming to counteract the development of sarcopenia is frequently advised by physicians, nevertheless few data are available on the targets and the global effects on the muscle organ of adapted exercise especially if started at old age.

**Methods:** To contribute answering this question for medical translational purposes, the proteomic profile of the gastrocnemius muscle was analyzed in 24-month-old mice undergoing adapted physical training on a treadmill for 12 weeks or kept under a sedentary lifestyle condition. Proteomic data were implemented by morphological and morphometrical ultrastructural evaluations.

**Results and Discussion:** Data demonstrate that muscles can respond to adapted physical training started at old age, positively modulating their morphology and the proteomic profile fostering protective and saving mechanisms either involving the extracellular compartment as well as muscle cell components and pathways (*i.e.,* mitochondrial processes, cytoplasmic translation pathways, chaperone-dependent protein refolding, regulation of skeletal muscle contraction). Therefore, this study provides important insights on the targets of adapted physical training, which can be regarded as suitable benchmarks for future *in vivo* studies further exploring the effects of this type of physical activity by functional/metabolic approaches.

## 1 Introduction

Physical activity is progressively reduced and/or impaired during aging causing mobility limitations and consequently a poor quality of life. In later stages, this decline may be associated to sarcopenia (Cruz-Jentoft et al., 2019; Chen et al., 2020), a muscle disease characterized by loss of skeletal muscle mass and strength associated with increased risk of frailty, falling and mortality (Cruz-Jentoft and Sayer, 2019). Many factors contribute to the progressive loss of muscle efficiency such as age, gender, comorbidities, body mass index, and malnutrition (Landi et al., 2013; Batsis et al., 2014; Beaudart et al., 2017; Robinson et al., 2018). Therefore, preventive issues and/or treatment of elderly require multidisciplinary approaches and a broad spectrum of competences.

A regular physical activity and/or exercise, performed throughout life, can slow down the age-dependent decline of muscle mass and strength, maintaining structure and function of neural and vascular compartments as well as muscles (Gao et al., 2020), and can prevent both age-associated muscle insulin resistance and inflammation (Novelli et al., 2004; Mikkelsen et al., 2013). However, lifelong physical training is a relatively rare situation in the general population, while physical exercise in adulthood or in the elderly for rehabilitation purposes (*e.g.,* after fractures or cardiovascular events) or simply to delay the effects of ageing is a rather frequent event. Therefore, it is important to understand if and how the muscle organ can respond to physical training started in old age. Furthermore, it is also important to take into consideration the intensity of exercise to avoid cardiovascular system overload opposing the widely described benefits of physical activity (La Gerche et al., 2017; Graziano et al., 2022). Within this context, adapted exercise, i.e., any modalities of physical training tailored on the residual physical capacity of the elderly in terms of frequency, intensity, and volume, was proposed as a way to optimize functional outcomes of physical exercise in both fit and frail older adults (Imagita et al., 2020; Izquierdo et al., 2021).

Investigations focusing on the role of physical activity in aged subjects indicate that it preserves fiber’s morphology, mitochondrial function (*i.e.*, ATP production) and antioxidant capacity (Proctor et al., 1995; Lanza et al., 2008; Safdar et al., 2010; Zampieri et al., 2015). However, these studies were mostly performed through reductionist-based approaches that, being restricted to specific molecular targets, can only improve the understanding of single biological mechanisms, without a global evaluation of the whole muscular microenvironment in response to physical activity and/or exercise.

In recent years, omic’s-based techniques (*e.g.,* transcriptomics, and proteomics) have been applied to explore the skeletal muscle behavior in different experimental conditions (Vissing and Schjerling, 2014; Lang et al., 2017; Starnes et al., 2017). The proteome profile of skeletal muscles has been investigated in aging (Doran et al., 2009; Gueugneau et al., 2014; Gonzalez-Freire et al., 2017; Murgia et al., 2017; Lofaro et al., 2021), and in metabolic diseases (Kleinert et al., 2018), as well as in response to high altitude hypoxia (Levett et al., 2015) and after interval, endurance or strength training (Holloway et al., 2009; Son et al., 2011; Schild et al., 2015; Petriz et al., 2017). Results clearly indicate that the response of skeletal muscles varies depending on the type and duration of the exercise (*e.g.,* few days vs*.* few weeks) (Egan and Zierath, 2013). However, it is still unclear whether the effects of physical training on the proteomic profile of skeletal muscle also occur when training is undertaken at old age.

To contribute answering this question, we analyzed the proteomics of the gastrocnemius muscle (GAST) in 24-month-old mice undergoing adapted physical training (treadmill running 3 days a week for 12 weeks, O-T) or kept in cages under a sedentary lifestyle condition (O-S). Proteomic data were further implemented by morphological and morphometrical ultrastructural evaluations.

Our findings demonstrate that: i) old muscles respond to adapted physical training started at old age, ii) adapted physical training positively modulates the morphology and the proteomic profile of GAST either involving the extracellular compartment as well as muscle cell components and pathways (*i.e.,* mitochondrial processes, cytoplasmic translation pathways, chaperone-dependent protein refolding, regulation of skeletal muscle contraction).

All these data emphasize that adapted physical training started at old age activates protective and saving mechanisms that act in synergy to counteract the age-dependent phenotype of GAST muscle.

## 2 Materials and methods

### 2.1 Mice

A total of 18 male BALB/c mice aged 24 months were used in this study. Male mice were chosen to avoid sex and hormonal status influence. The mice, housed in groups of 3–4, were maintained under standard conditions (24° ± 1°C ambient temperature, 60% ± 15% relative humidity, and 12 h light/dark cycle) and fed *ad libitum* with standard commercial chow. The mice were allocated to the sedentary old (O-S, *n* = 8) and training old (O-T, *n* = 10) group with the “ = Rand()” function in Microsoft Excel. O-S mice were only allowed only spontaneous free-moving activity in the cage. O-T mice underwent training on a treadmill (Harvard Instruments, Crisel, Rome, Italy) for 30 min at 8 m/min belt speed (0% incline), 3 days a week for 12 weeks. Running on a treadmill is a widely used training modality in laboratory animals because it allows for accurate control and extensive modulation of exercise intensity, duration, and frequency, making it suitable even for older individuals (Massett et al., 2021). Current treadmill protocols for adult individuals consistently use 1 h running a day at belt speed >10 m/min. In this work, physical training was adapted to optimize old mice compliance to training (Fabene et al., 2008). To minimize stress several provisions were adopted. Running was continuously supervised by an experimenter. Mice familiarized for 1 week to the treadmill with a single 30-min daily session on the stopped treadmill. During the experiment, mice were transported to the running room 1 h before each daily session of exercise. According to the standard protocol of our laboratory to prevent injury to the hind limbs provoked by the posterior wall of the treadmill a metal-beaded curtain, as non-noxious stimulus, was used as an incentive; no shock-plate incentive was used. To avoid possible interference of acute with chronic effects of physical exercise, the mice were sacrificed 3 days after completion of the experimental training protocol. Animals were deeply anesthetized with tribromoethanol drug and sacrificed by cervical dislocation or perfused via the ascending aorta with 0.1 M PBS followed by 4% paraformaldehyde in PBS depending on the analyses to be performed.

Evaluation of the results and data analysis were carried out blind to the mouse group.

Mice were handled according to the regulations of the Italian Ministry of Health (DL 4 March 2014, n. 26) and to the European Communities Council (Directive 63/2010/EU of the European Parliament and the Council) directives. The experimental protocol was approved by the Italian Ministry of Health (Ref.: 538/2015-PR).

The gastrocnemius muscle was selected for this study since it is one of the primary movers of the hindlimb, being involved in gait; moreover, it is prevalently composed of fast-twitch fibers (Zancanaro et al., 2007), which are especially affected by atrophy during aging (Lexell, 1995).

### 2.2 In tube-gel digestion

For proteomic analysis, whole gastrocnemius muscles were quickly removed from three O-S and three O-T (sacrificed by cervical dislocation) and frozen.

Tissue protein digestion was performed as previously described (Lofaro et al., 2021). Briefly, three subsequent extraction protocols were used to isolate proteins with different solubility grade. Therefore, frozen muscles were homogenized in phosphate buffer (PBS) on ice using a glass homogenizer to solubilize cellular and hydrophilic proteins. The resulting homogenates were then centrifuged at 8,000× g for 30 min at 4°C. The supernatants (PBS extract) were collected, whereas pellets were resuspended in urea-thiourea buffer and incubated at 4°C for 24 h with continuous shaking to favor the solubilization of hydrophobic proteins. The samples were centrifuged at 150,00× g for 20 min at 4°C to obtain the supernatant (U/T extract), while the pellet was homogenized in guanidinium-HCl buffer (pH = 8.5, 1:5 w/v), heated at 100°C for 10 min, and collected (GuHCl extract). This last extraction is considered the most appropriate for insoluble ECM proteins. The protein concentration of each fraction was determined using the Bradford method (Bradford, 1976).

For each fraction (200 µg of proteins), a gel-tube digestion method was performed, as already described (Boraldi et al., 2019). The proteins embedded in the gel-tubes were firstly reduced by incubation with 10 mM dithioerythritol and 100 mM ammonium bicarbonate at 56°C for 45 min. Subsequently, they were alkylated with 55 mM iodoacetamide and 100 mM ammonium bicarbonate for 30 min at room temperature in the dark. Protein digestion was performed by trypsin with an enzyme-to-protein ratio of 1:100 (Promega, Madison, MI, United States). Peptides were extracted from the gel-tubes using 100% acetonitrile and subsequently dried in a SpeedVac (Eppendorf AG, Hamburg, Germany).

### 2.3 LC–MS/MS analysis and MS/MS data processing

Mass spectrometric analysis was performed using an LC coupled to a QExactive mass spectrometer (Thermo Fisher); MS/MS data (.raw) were examined using BatMass (v. 0.3.0), as previously described (Lofaro et al., 2021). Briefly, replicates were aligned using FreeStyle (v.1.5) to assess the run quality. The raw files were then converted to. mzXML format using msConvert ProteoWizard (v.3.0.19239) with default settings and uploaded to the MSFragger (v.3.4.0) for MS/MS Ion Search. The search was conducted using the Uniprot database (2018_05) limited to *Mus musculus* (Taxonomy ID: 10090). Enzyme was specified to trypsin with a maximum of 1 missed cleavage. Fix modification was carbamidomethylation of cysteine, whereas variable modifications were deamidation of asparagine and glutamine, oxidation of methionine, and cysteine propionamide. Data searches were performed with a mass error tolerance of 10 ppm for precursor ions and of 0.02 Da for fragment ions with peptide charge states of 2+, 3+, and 4+.

Only confidently identified peptides with a false discovery rate (FDR) ≤ 1 and proteins with at least one unique peptide were exported.

The MSFragger results (interact.pep) obtained for each extraction were imported into Skyline-daily (v.21.0.9.139) to generate spectral libraries using the following parameters: a spectra cut-off score of 0.95, peptide length ranging from 8 to 25 amino acids, and precursor ion charges of 2+, 3+, and 4+. MS1 filters were set to “use high selectivity extraction” with a resolving power of 60,000 at 300 m/z, and repeated and duplicate peptides were removed. Following the Skyline “DDA peptide search” workflow, the raw files (.raw) were imported and matched to the spectral libraries to recover precursor ion intensity. Precursor ion intensity represents the sum of areas under the curve of extracted ion chromatograms (XICs) containing precursor ion isotope peaks (M, M+1, M+2) (Singhto and Thongboonkerd, 2018). Fasta files containing proteins with a 1% FDR were imported into Skyline to maintain and fix the FDR.

The quantitative analysis was only conducted on proteins identified with at least two peptides because the quantification of a single peptide across LC-MS/MS runs could be not sufficiently accurate (Tsai et al., 2020). Statistical analysis of proteomic data was performed with the Skyline group comparison tool. Runs were normalized using the “Equilize Medians” method with a confidence level of 0.95, and the Tukey’s Median Polish was used as the summary method (Lofaro et al., 2021).

### 2.4 Protein-protein interaction (PPI) analyses

PPIs were built by STRING database (v. 11.5) to understand functions and interactions of DEPs (Szklarczyk et al., 2021). Furthermore, MCL clustering with a minimum of 3 inflation parameters was employed to identify the top 5 protein clusters, which were imported and edited into Cytoscape (v.3.9.1) to visualize networks.

### 2.5 Immunofluorescence

For identification of diffuse and tissue-abundant antigens, we adopted an immunohistochemical approach, briefly described below.

#### 2.5.1 Laminin

GAST muscles from at least three O-S and three O-T mice were frozen in liquid nitrogen-precooled isopentane, transversally sectioned into 5-µm thick cryosections and incubated with 1% bovine serum albumin, 2% normal goat serum, 0.3% Triton^®^ X-100 in PBS for 1 h and immunolabelled with a rabbit polyclonal antibody direct against laminin, diluted 1:800 (Abcam, Cambridge, United Kingdom). After washing with PBS, cryosections were stained with the secondary antibody Alexafluor 594-anti-rabbit diluted 1:200. The cryosections were finally counterstained for DNA with 0.1 mg/mL Hoechst 33,258 and mounted in PBS:glycerol (1:1). An Olympus BX51 microscope equipped with a 100 W mercury lamp (Olympus Italia, Milan, Italy) was used under the following conditions: 540 nm excitation filter (excf), 580 nm dichroic mirror (dm), and 620 nm barrier filter (bf) for Alexa 594; 330–385 nm excf, 400 nm dm, and 420 nm bf for Hoechst 33,258. Images were recorded with an Olympus Magnifire digital camera system (Olympus Italia). In anti-laminin immunolabeled samples, the minimum Feret’s diameter (the minimum distance of parallel tangents at opposing borders of the muscle fibers) was measured on 200 myofibers per mice group. The minimum Feret’s diameter is very insensitive against deviations from the “optimal” cross-sectioning profile, therefore, reliably detecting differences between muscles (Briguet et al., 2004).

#### 2.5.2 Heavy chain of skeletal fast fiber myosin

GAST samples (about 1 mm^3^) from perfused animals (three O-S and three O-T mice) were placed for 2 h at 4°C in 4% paraformaldehyde and 0.2% glutaraldehyde in 0.1 M PBS, washed in PBS, treated with 0.5 M NH_4_Cl solution in PBS for 45 min at 4°C to block free aldehyde groups, dehydrated in graded concentrations of ethanol at room temperature and embedded in LRWhite resin. For fiber typing, 2 μm-thick cross sections were submitted to immunohistochemical procedures to distinguish fast and slow fibers (Egan and Zierath, 2013). Briefly, sections were incubated for 2 h at room temperature with a mouse monoclonal antibody recognizing the heavy chain of skeletal fast fiber myosin (clone MY-32, Sigma-Aldrich, Buchs, Switzerland) diluted 1:200 in PBS; the antigen–antibody complex was revealed with an Alexa 488 conjugated antibody against mouse IgG (Molecular Probes, Invitrogen, Milan, Italy). The sections were finally counterstained for DNA with 0.1 μg/mL Hoechst 33,258. Micrographs were taken with an Olympus BX51 microscope equipped with a 100 W mercury lamp under the following conditions: 450- to 480-nm excitation filter (excf), 500-nm dichroic mirror (dm), and 515-nm barrier filter (bf) for Alexa 488; 330- to 385-nm excf, 400-nm dm, and 420-nm bf for Hoechst 33,258. Images were recorded with an Olympus Camedia C-5050 digital camera. In immunolabeled samples, the percentage of fast and slow muscle fibers was calculated on 100 myofibers per animal, with 300 myofibers measured per group (O-S and O-T). Micrographs were taken at magnification of ×20 and processed with the ImageJ software (NIH).

### 2.6 Ultrastructure and morphometric evaluations

GAST samples (about 1 mm^3^) from perfused animals (three O-S and three O-T mice) were placed for 2 h at 4°C in 2.5% glutaraldehyde (Electron Microscopy Sciences, Hatfield, PA, United States) plus 2% paraformaldehyde. After fixation, samples for ultrastructural morphology were rinsed with PBS, postfixed with 1% OsO_4_ (Electron Microscopy Sciences) and 1.5% potassium ferrocyanide for 2 h at 4°C, dehydrated with acetone, and embedded in Epon 812 resin (Electron Microscopy Sciences).

For morphometrical evaluation of ultrastructural variables, ultrathin sections (70–90 nm thick) from three O-S and three O-T mice were stained with lead citrate for 1 min and observed in a Philips Morgagni transmission electron microscope operating at 80 kV and equipped with a Megaview III camera for digital image acquisition.

The morphometric evaluation of the endomysium thickness was performed on 20 randomly selected electron micrographs (×5,600) of longitudinally sectioned muscle. The distance between the sarcolemma of two adjacent muscle cells was measured every 1 µm of sarcolemma length, for a total of 50 measurements per animal with a total of 150 measurements for mice group (O-S and O-T). The thickness of the basement membrane covering the myofiber was measured on randomly selected electron micrographs (×36,000) of longitudinally sectioned muscles. A minimum of 30 measurements per animal were performed for a total of 100 measurements for mice group (O-S and O-T).

The morphometric evaluation of the perimysium collagen bundle size was performed on 25 longitudinally sectioned collagen bundles per animal, for a total of 75 measurement for each mice group (O-S and O-T). The index of collagen bundle linearity (X/Y, expressed as the ratio between the real length of the bundle profile and the corresponding linear length) was assessed on 10 longitudinally sectioned collagen bundles per animal, for a total of 30 measurements per mice group (O-S and O-T). For morphometric evaluation of collagen fibrils, measurement of fibril size, as well as the distance between single collagen fibrils, was performed on a minimum of 30 longitudinally sectioned collagen fibrils per animal with a total of 100 measurements per mice group (O-S and O-T).

The density of the perinuclear and intermyofibrillar mitochondria was assessed in 10 randomly selected micrographs (×11,000) of longitudinally sectioned muscle per animal for a total of 30 measurements per mice group (O-S and O-T). For the assessment of perinuclear mitochondria density, the cytoplasmic area surrounding myonuclei and devoid of myofibrils bundles was considered. For the intermyofibrillar mitochondria density, cytoplasmic areas far from the myofiber periphery were considered. The number of mitochondria was counted, and the mitochondrial density was expressed as number of mitochondria/area (µm^2^).

The sectional area of both intermyofibrillar and perinuclear mitochondria as well as the length of outer and inner mitochondrial membrane was measured in 20 mitochondria (×36,000) per animal for a total of 60 mitochondria per mice group (O-S and O-T). The inner/outer membrane ratio, an assessment of cristae extension independent of mitochondrial size, was calculated as a reliable morphological parameter of mitochondrial function (*i.e.,* respiratory activity). In fact, the cristae density is considered a predictor of maximum oxygen uptake in relation to mitochondrial volume (Nielsen et al., 2017).

Morphometrical analysis of the nucleolus and the nucleolar components, *i.e.,* fibrillar centers (FCs, circular in shape, contain ribosomal genes and enzymes necessary for transcription (Recher et al., 1969; Goessens, 1984), dense fibrillar component (DFC, usually edges the FCs, is the site of transcription and processing of rRNA (Schwarzacher and Wachtler, 1991) and granular component (GC, site of maturation and storage of ribosomal subunit (Schwarzacher and Wachtler, 1991), was performed on 10 randomly selected nucleoli of longitudinally sectioned muscles (×18,000) per animal for a total of 30 measurements per mice group (O-S and O-T). Area of the nucleolus as well as each nucleolar component were measured, and the percentage of the nucleolar areas occupied by FCs, DFC, and GC was calculated.

All measurements were made by using the Radius software for image acquisition and elaboration implemented in the Philips Morgagni transmission electron microscope.

### 2.7 Ultrastructural immunocytochemical analyses

For identification of sub-cellular and less abundant antigens, we adopted an ultrastructural, immune-electron microscopy approach allowing for more accurate and sensitive antigen localization in spite of the less intense signal compared to light microscopy immunohistochemistry.

GAST samples (about 1 mm^3^) from perfused animals (three O-S and three O-T mice) were placed for 2 h at 4°C in 4% paraformaldehyde and 0.2% glutaraldehyde in 0.1 M PBS, washed in PBS, treated with 0.5 M NH_4_Cl solution in PBS for 45 min at 4°C to block free aldehyde groups, dehydrated in graded concentrations of ethanol at room temperature and embedded in LRWhite resin. This procedure, while providing poorer morphology vs*.* standard embedding (*e.g.,* epossidic resin), makes samples much more suitable for antigen detection.

Longitudinally-cut sections (70–90 nm thick), collected on Formvar-carbon-coated nickel grids, were floated on normal goat serum (NGS) diluted 1:100 in PBS for 3 min and then incubated at 4°C for 17 h with a polyclonal antibody directed against telethonin (sc-8725, Santa Cruz biotechnology, Dallas, United States) diluted 1:25 or a monoclonal antibody directed against mitochondrial hsp70 (mtHsp70; Enzo Life Sciences, Farmingdale, NY, United States) diluted 1:100, both diluted in PBS containing 0.05% Tween and 0.1% BSA. After rinsing with PBS, Tween and PBS, the grids were incubated with NGS as above, and then incubated for 30 min at room temperature with the appropriate secondary antibody (Jackson Immuno Research Laboratories, West Grove, PA, United States) coupled with colloidal gold, diluted 1:20 in PBS. The grids were rinsed with PBS and distilled water. Sections were stained for 35 min at room temperature with Uranyl Less EM stain (Electron Microscopy Sciences, Hatfield, PA, United States), followed by Reynolds’ lead citrate for 1 min (Lacavalla and Cisterna, 2023). Sections were then observed with a Philips Morgagni transmission electron microscope operating at 80 kV.

As controls, some grids were floated on the incubation mixture without the primary antibody, and then treated as above. Immunolabelling was absolutely negligible.

A semiquantitative assessment of the anti-telethonin immunolabeling was carried out by estimating the gold grain density on 10 myofiber area for a total of 30 myofiber area per each mice group. The gold grains were counted, and the labelling density was expressed as number of gold grains/myofiber area (µm^2^).

A semiquantitative measurement of anti-mithsp70 immunolabelling was carried out on a minimum of 10 intermyofibrillar mitochondria per animal for a total of 40 measurements per mice group. The gold grains were counted, and the labelling density was expressed as number of gold grains/mitochondrial area (µm^2^).

### 2.8 Statistical analysis

Quantitative and semiquantitative values for individual variables were pooled according to the experimental group (O-S and O-T) and are presented as mean ± standard error of the mean (SEM). Between-group comparison of body mass was carried out with the *t*-test for independent samples (O-T vs*.* O-T at baseline; GAST mass at endpoint; nucleolar area at endpoint) or the *t*-test for paired samples (O-S and O-T before and after training). For all other variables, the Shapiro-Wilk test showed non normal distribution of the residuals of the regression of the variable on mouse ordinal number (intended as a categorical variable); therefore, the non-parametric Mann–Whitney test was used throughout. The statistical significance was set at *p*-value < 0.05. The IBM-SPSS (v.25, Armonk, NY, United States) statistical package was used for all analyses.

## 3 Results and discussion

### 3.1 Effects of adapted physical training on mice

All mice in the O-T group were able to correctly carry out the physical training protocol (see *materials and methods*).

At baseline, body mass was similar in the two groups of mice (O-S, 39.7 ± 1.92 g; O-T, 39.7 ± 0.76 g; p = N.S.). Body mass showed statistically significant decrease in O-S at the end of the training period (34.8 ± 1.37 g, t = 3.954, *p* = 0.011), whereas it was not statistically significant different in O-T (37.8 ± 1.58 g; t = 1.031, *p* = 0.350). These findings indicate that adapted physical training contributes preventing age-related weight loss. At the end of the training period the average mass of GAST was slightly (*p* = 0.21) higher in O-T (170 ± 20 mg) than O-S (160 ± 17 mg).

### 3.2 Effects of adapted physical training on the protein profile of GAST

By using three different extraction buffers (Lofaro et al., 2021) (see materials and methods) followed by LC–MS/MS analyses, 1,493 different proteins were identified in aged mice (Supplementary Table S1), of which 369 were present in all 3 extracts, while 416, 182, and 51 proteins were detected in phosphate buffered saline (PBS), urea and thiourea (U/T) and guanidinium-HCl (GuHCl) extracts, respectively. A label free quantification was applied to the proteins identified with at least two peptides (Supplementary Table S2).

**FIGURE 1:**
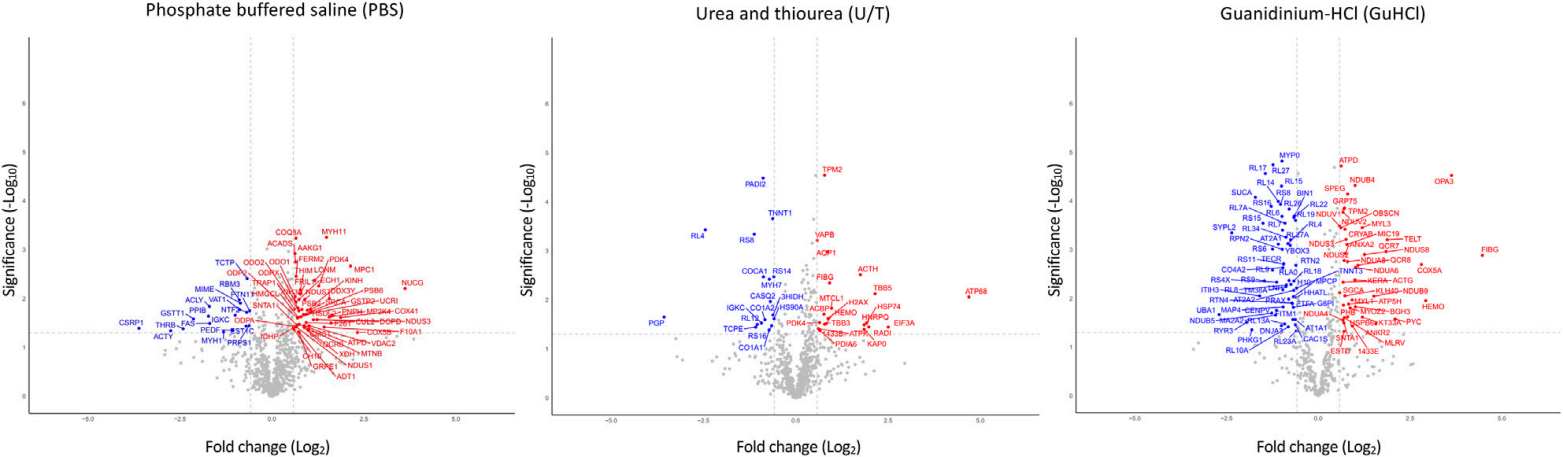
Volcano plots of differentially expressed proteins. Analysis was performed on the three fractions resulting from various extraction procedures. Non-significantly (grey) and significantly down- (blue) and upregulated (red) proteins in treadmill training (O-T) vs. sedentary (O-S) mice (with fold change <2 or >2) are shown. *p*-value was set to match q-value < 0.05.

Figure 1; Supplementary Table S3 show the differentially expressed proteins (DEPs) between O-T and O-S mice in each extract: 50 and 18 proteins were up- or downregulated in PBS fraction; 21 and 17 were up- or downregulated proteins in U/T fraction and 43 and 60 proteins were up- or downregulated in GuHCl fraction. The use of subsequent different extraction procedures allowed to markedly increase the number of identified proteins compared, for instance, to similar proteomic studies (Li et al., 2019).

Taken together, these results show that a total of 197 proteins were significantly modified by adapted physical training.

### 3.3 Pathway enrichment analysis

**FIGURE 2:**
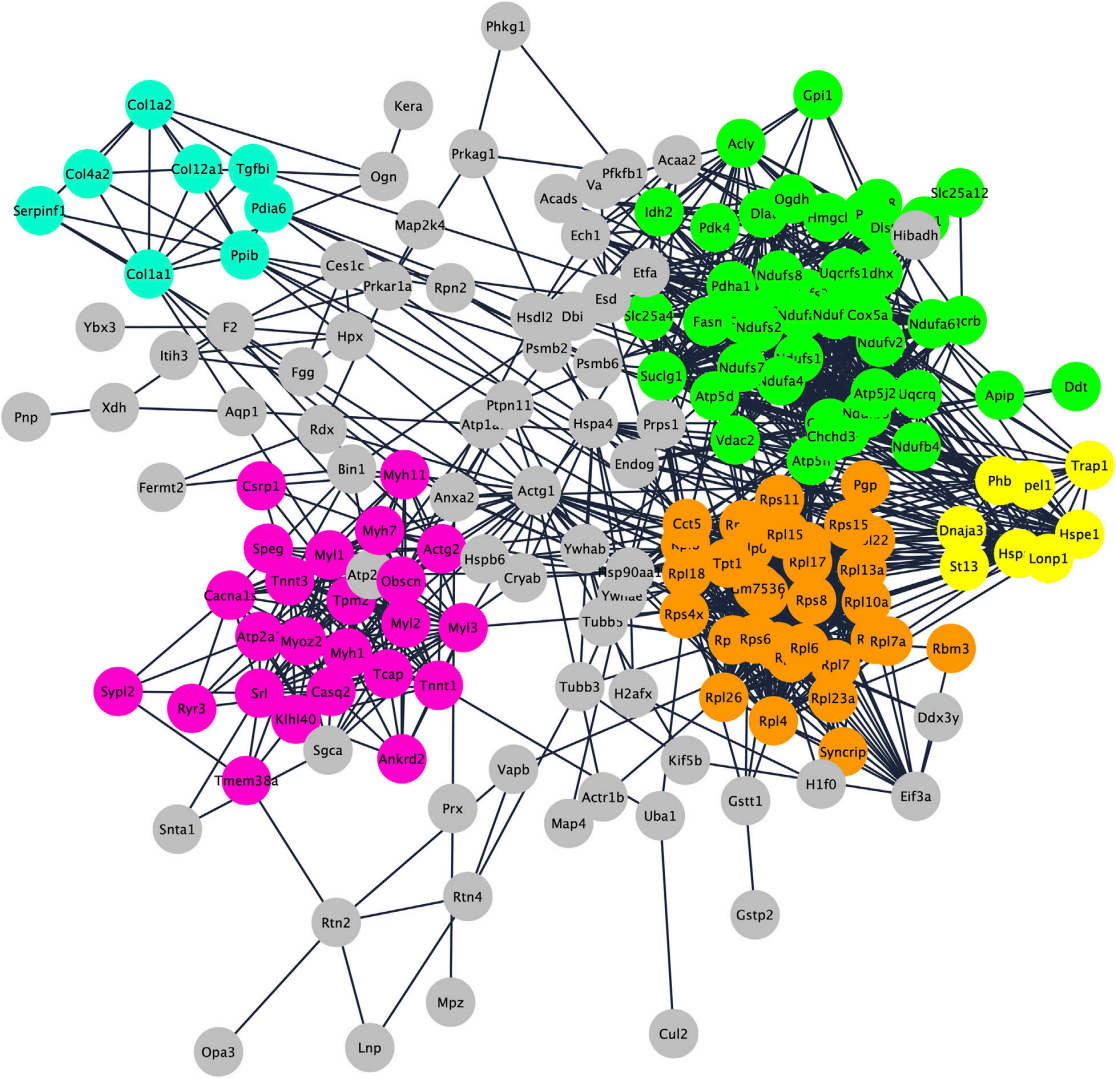
Protein–protein interaction and enrichment analysis of 192 DEPs obtained by STRING. Networks (clusters) with 8 or more proteins (densely connected nodes) were colored in green (cluster 1; mitochondrial processes), orange (cluster 2; cytoplasmic translation pathways), pink (cluster 3; regulation of skeletal muscle contraction), light blue (cluster 4; chaperone-dependent protein refolding) and yellow (cluster 5; collagen-activated signaling pathways).

To understand functions and interactions of DEPs, we used the STRING database, version 11.5 (Szklarczyk et al., 2021). Figure 2 shows the five different subnetworks that were identified by STRING according to the protein-protein interaction (PPI) network of DEPs. These clusters were enriched in proteins associated with mitochondrial processes (cluster 1; GO:0061732); cytoplasmic translation pathways (cluster 2; GO:0002181); regulation of skeletal muscle contraction (cluster 3; GO:0014724); chaperone-dependent protein refolding (cluster 4; GO:0051085); collagen-activated signaling pathways (cluster 5; GO:0038063). These data clearly indicate that DEPs are not scattered in the secretome but are mostly connected within specific pathways/functions. Disclosing protein-protein interactions through the interactome may contribute to understand the cell process-regulating machinery and to pave the way for the identification of potentially new biomarkers (Feng et al., 2015).

### 3.4 Effects of adapted physical training on mitochondria processes and cytoplasmic translation pathways

Skeletal muscle contractile activity affects mitochondrial and ribosome biogenesis (Mesquita et al., 2021), however studies revealed apparently opposing results depending on the training program (*e.g.,* endurance vs*.* resistance; endurance followed by resistance or vice versa; frequency and/or duration of exercise). Exercise, in fact, can simultaneously influence ribosome and mitochondrial biogenesis, or can increase ribosome biogenesis over mitochondrial biogenesis or vice versa as a consequence of the competition between the two processes (Morrison et al., 1989; Wilkinson et al., 2008; Gibala et al., 2009; Hansson et al., 2019; Figueiredo et al., 2021).

**FIGURE 3:**
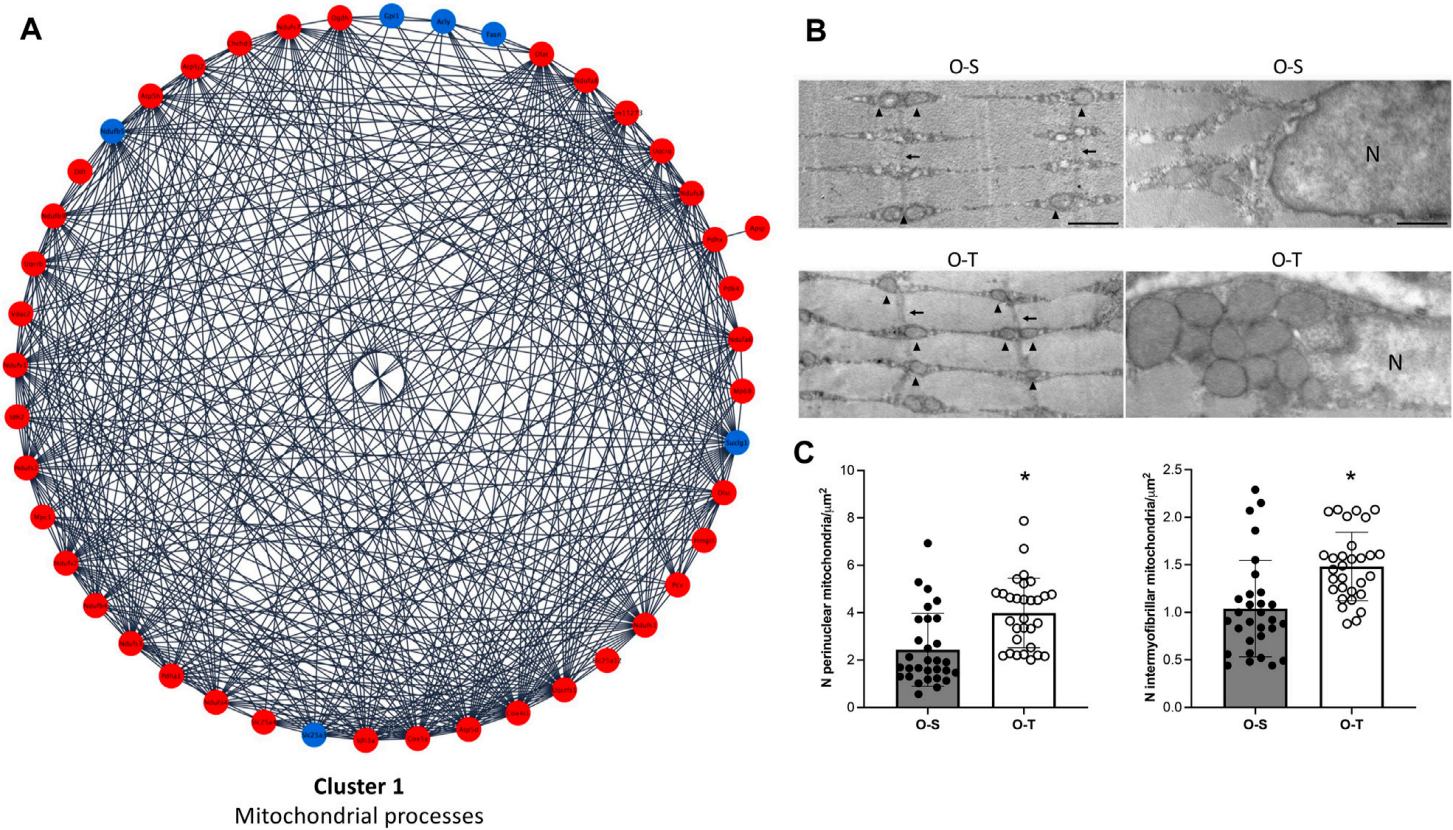
Mitochondria proteomics and ultrastructure. **(A)** Differential expression of proteins belonging to cluster 1 showing up- (red) and down- (blue) regulated proteins in mice undergoing adapted physical training compared to sedentary animals. **(B)** Intermyofibrillar (left) and perinuclear (right) mitochondria in representative ultrastructural images of gastrocnemius myofibers from old sedentary (O-S) and old trained (O-T) mice. Intermyofibrillar mitochondria are indicated by arrowheads and Z-lines by thin arrows. Bars: 500 nm. N = nucleus **(C)** The mitochondrial density is expressed as number of mitochondria per myofiber area far from myofiber periphery (intermyofibrillar mitochondria) or per cytoplasmic area surrounding myonuclei (perinuclear mitochondria). Scatter dot plots show individual values, mean ± SD. *p < 0.001.

**FIGURE 4:**
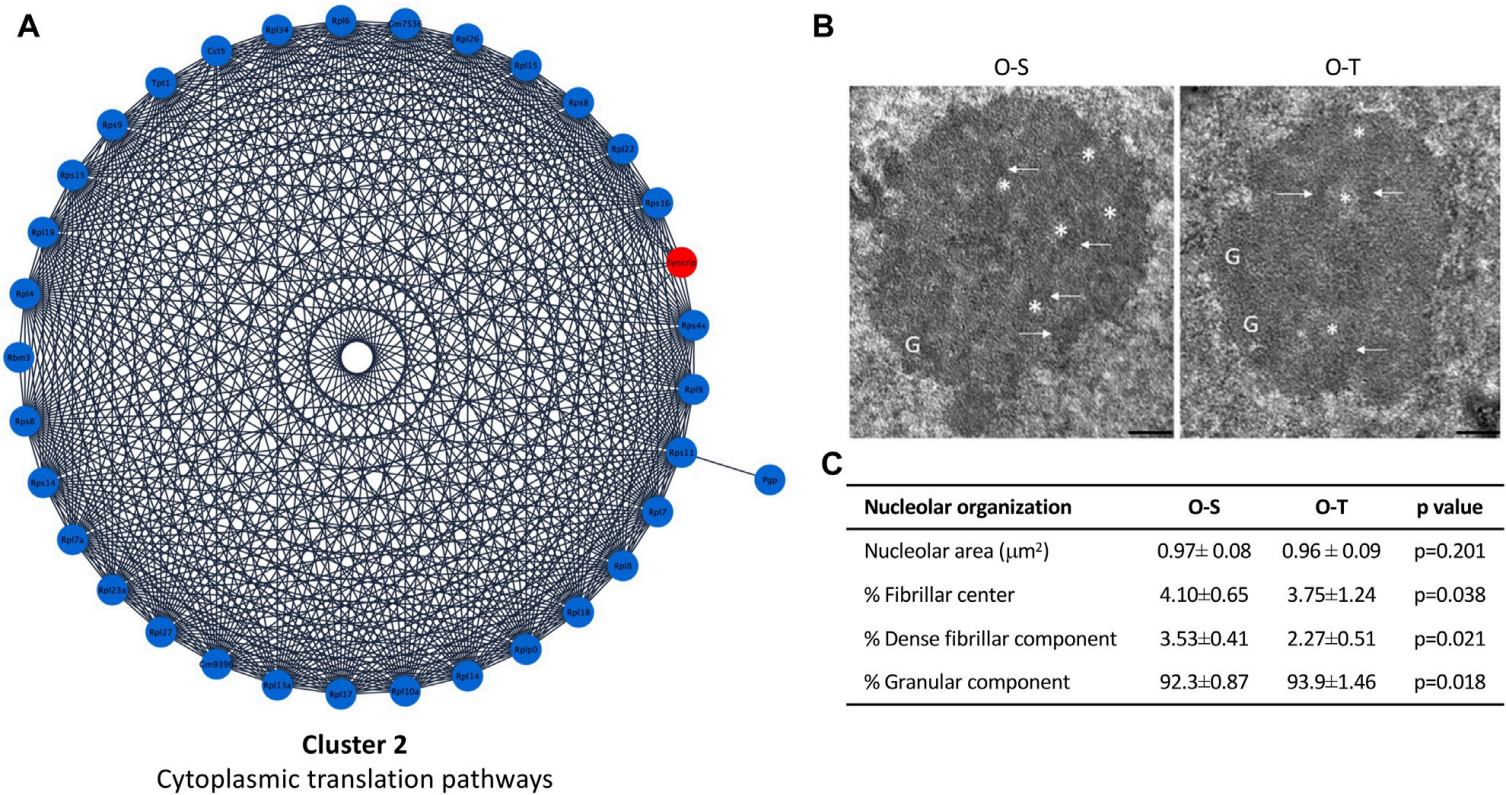
Cytoplasmic translation pathways and nucleolar ultrastructure. **(A)** Differential expression of proteins belonging to cluster 2 showing up- (red) and down- (blue) regulated proteins in mice undergoing adapted physical training compared to sedentary animals. **(B)** Ultrastructural images of nucleolus of old sedentary (O-S) and old trained (O-T) mice. Bars: 200 nm. Dense fibrillar component (arrow), fibrillar center (asterisk) and granular component (G) are shown. **(C)** The nucleolar area, the percentage of nucleolar areas occupied by fibrillar center, dense fibrillar component and granular component in O-S and O-T are reported.

In our experimental conditions we found that: i) 39 and 6 polypeptides related to mitochondrial processes were up- or downregulated by physical training, respectively (Figure 3A; Supplementary Table S3), whereas ii) 33/34 DEPs related to ribosomes were downregulated after the training period (Figure 4A; Supplementary Table S3).

Therefore, adapted long-term treadmill running increased the level of mitochondrial proteins, many of which belong to the mitochondrial complex I, a multimeric enzyme that modulates the transfer of electrons from NADH to ubiquinone, facilitating ATP synthesis (Galkin et al., 2006). These data are in agreement with those observed in the rat model (Li et al., 2019), thus further highlighting the role of mitochondria metabolism/activity as a preferential target of physical training, although the extent of these changes may differ according to the age and/or to the type of exercise. Present data demonstrate that mitochondria are still responsive in aged animals.

Since it has been demonstrated that structure and function are tightly connected and that mitochondrial morphology is strictly linked with changes in energy production (Buffa et al., 1970; Muscatello and Pasquali-Ronchetti, 1972; Glancy et al., 2020; Lofaro et al., 2020), the ultrastructure of myofibers and of mitochondria was investigated.

The typical organization of the myofibers was maintained in both O-S and O-T. Many myonuclei were in subsarcolemmal position and the longitudinally aligned myofibrils occupied almost the entire cytoplasm. Cisternae of sarcoplasmic reticulum, often surrounded by abundant glycogen deposits, were distributed between the myofibrils. Few lipid droplets were found in the myofibers. Well preserved ovoid mitochondria were located in the subsarcolemmal region. The great majority of mitochondria were lined between the myofibrils (Figure 3B), consistently with the role of intermyofibrillar mitochondria that are the site of biochemical pathways related to muscle contraction (Müller, 1976). The density of both intermyofibrillar and perinuclear mitochondria was significantly higher in O-T vs*.* O-S (Figure 3C). No significant difference was found in the size of the intermyofibrillar mitochondria in O-S vs*.* O-T (0.084 ± 0.009 µm^2^ vs*.* 0.076 ± 0.007 µm^2^; *p* = 0.68) as well as in the mitochondrial cristae extension, a reliable morphological parameter of mitochondrial function (Nielsen et al., 2017) (1.206 ± 0.055 vs*.* 1.233 ± 0.082; *p* = 0.44). The mitochondrial size and the cristae extension were instead significantly higher in perinuclear mitochondria of O-T vs*.* O-S (0.137 ± 0.017 vs*.* 0.055 ± 0.004 µm^2^, *p* < 0.001 and 1.615 ± 0.111 vs*.* 1.286 ± 0.069, *p* = 0.01, respectively).

The significant increase of mitochondrial density in trained mice is in agreement with data indicating that in aged skeletal muscle, the decreased mitochondrial density as well as the reduced respiratory activity are mostly related to decreased physical activity (Rasmussen et al., 2003; Russ and Kent-Braun, 2004; Distefano et al., 2018). Therefore, it can be hypothesized that the higher density of both intermyofibrillar and perinuclear mitochondria as well as the increased size and cristae extension of the latter may lead to increased ATP availability (Glancy et al., 2015; Glancy and Balaban, 2021; Willingham et al., 2021).

These findings also suggest that the perinuclear mitochondrial population is more sensitive to the stimulating effect of physical training in the skeletal muscle of old mice.

In our model, ribosome proteins were significantly downregulated after training (Figure 4A; Supplementary Table S3), suggesting a general reduction/delay of protein synthesis, although some proteins/pathways may be specifically upregulated as shown by analyses of DEPs. Indeed, these data are consistent with the observation that reduced protein synthesis allows to increase lifespan (Tavernarakis, 2007; 2008), since it may represent a saving mechanism reducing the risk of accumulating damaged proteins (Hipkiss, 2007), thus avoiding the overloading of the protein quality control system, and the development of a pro-aging environment (López-Otín et al., 2013).

Moreover, protein synthesis is one of the most energy-consuming cellular processes (Lane and Martin, 2010), therefore depletion of ribosomal proteins and of translational factors can increase stress resistance allowing the switch to the selective production of proteins required not only to sustain muscle contraction, but also to foster cellular maintenance, repair, and turnover pathways (Gonskikh and Polacek, 2017).

To assess whether an adapted physical exercise affects protein synthesis by influencing production and/or export of ribosomal subunits, morphometrical evaluation of the nucleolar area as well as the percentage of nucleolar areas occupied by fibrillar center (FC, where rDNA is located), dense fibrillar component (DFC, site of transcription and processing of rRNA, and assembly of the ribosomal subunits) and granular component (GC, site of maturation and storage of ribosomal subunits) was carried out (Figure 4B). No significant difference in nucleolar area was found in O-T vs*.* O-S. On the contrary, the percentage of the nucleolar areas occupied by FC and DFC was significantly lower in O-T vs*.* O-S (Figure 4C), thus suggesting a decreased production of pre-ribosomes and, hence, decreased ribosomal protein requirement. Moreover, the observed downregulation of ribosomal proteins, concomitant with the enlargement of GC (Figure 4C), suggests a delay in the processing and release of the pre-ribosomes (Jamison et al., 2010). Evidence demonstrated that high rates of rDNA transcription may lead to genome instability and to accumulation of rDNA damage, which are typical features of cellular aging (Tsekrekou et al., 2017). Therefore, our results are consistent with the occurrence of protective mechanisms in trained old mice.

### 3.5 Effects of adapted physical training on the regulation of skeletal muscle contraction

**FIGURE 5:**
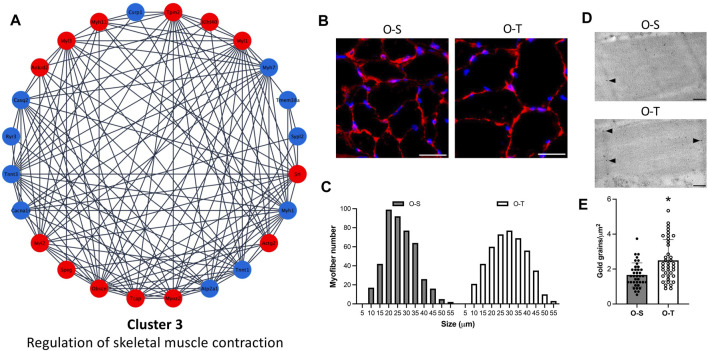
Contractile muscle system. **(A)** Differential expression of proteins belonging to cluster 3 showing up- (red) and down- (blue) regulated proteins in physically trained compared to sedentary mice. **(B)** Representative images of myofibers from old sedentary (O-S) and old trained (O-T) mice immunolabelled for laminin (red); DNA was counterstained with Hoechst 33,258 (blue). Bars: 100 μm. **(C)** Minimum Feret’s diameter distribution of the myofibers in the GAST muscle of O-S and O-T mice. Myofibers are grouped in size classes of 5 μm and the number of fibers in each class is plotted. **(D)** Telethonin immunolabelling on aldehyde-fixed and LRWhite-embedded samples. Representative ultrastructural images of telethonin immunolabeling (arrowheads) on Z-lines in old sedentary (O-S) and old trained (O-T) mice. Bar: 200 nm. **(E)** Quantitative evaluation of anti-telethonin immunolabelling per myofiber unit area in O-S and O-T mice. Scatter dot plot shows individual values, mean ± SD. *p < 0.001.

In the present study, adapted physical training in old mice was associated to remarkable modifications in the contractile muscle system (Figure 5A) characterized by both a reduction of Myh7 (log_2_Fold change O-T/O-S = −0.7131) and Tnnt1 (log_2_Fold change O-T/O-S = −0.623) and an increase of Myoz2 (log_2_Fold change O-T/O-S = 0.8695), Myl1 (log_2_Fold change O-T/O-S = 0.9223), Myl2 (log_2_Fold change O-T/O-S = 0.7101) and Myl3 (log_2_Fold change O-T/O-S = 1.1973) (Supplementary Table S3). These findings indicate a reorganization of the skeletal muscle contraction system in O-T compared with O-S mice.

According to data in the literature, in response to physiological and/or pathological signals, muscle fibers can reforge their phenotype to the environmental demand (*i.e.,* switch from fast to slow fibers and vice versa) (Bassel-Duby and Olson, 2006; Talbot and Maves, 2016). During aging a fast to slow fiber transition can be observed (Ohlendieck, 2011) and exercise may cause changes in the composition of muscle fiber types depending on the type/frequency of exercise and on the type of muscle (Lee et al., 2022).

Interestingly, in our experimental model, immunolabeling for the heavy chain of skeletal fast fiber myosin (Supplementary Figure S1) revealed no statistically significant difference in the percentage of fast myofibers in O-S vs*.* O-T (98.28 ± 1.21 vs*.* 99.75 ± 0.25, *p* = 0.47), with a negligible percentage of slow fibers in both mice groups.

Moreover, it is well known that aging is characterized by a progressive reduction in both muscle mass and myofiber size and that physical training can prevent muscle functional deficiency (Zancanaro et al., 2007). In the present study, to determine the size of myofibers, the sections of GAST muscle were immunolabelled with laminin, a basal lamina protein that individually outlines myofibers, and whose expression was not affected by adapted physical training (Supplementary Table S2). We observed an increase of the size of myofibers in O-T vs*.* O-S (Figure 5B), confirmed by the minimum Feret’s diameter distributions (Figure 5C) showing that more myofibers were in higher size classes in O-T vs*.* O-S with mean values of 26.74 ± 0.47 µm vs*.* 24.23 ± 0.41 µm (*p* < 0.001).

It is well known that increase of fiber cross-sectional area is accompanied by a proportional increase of myofibrillar proteins that are essential for the generation and propagation of mechanical forces and are under the control of regulatory components (*e.g.,* titin, obscurin and telethonin).

Interestingly, regulatory proteins (*i.e.,* ankyrin, obscurin, telethonin) were all upregulated in O-T mice (log_2_Fold change O-T/O-S = 1.5712, 0.6121, and 1.8802, respectively) (Figure 5A; Supplementary Table S3) and immunolabelling confirmed the upregulation of telethonin in O-T compared to O-S mice (Figures 5D, E).

Data from the present study indicate that adapted physical training started at old age is able to counteract the age-associated decrease of myofibers’ size, a typical feature of skeletal muscle aging (Zancanaro et al., 2007), and to upregulate the expression of regulatory proteins supporting sarcolemmal integrity (Subramaniam et al., 2022). Instead, physical training did not affect the percentage of slow fibers in GAST.

### 3.6 Effects of adapted physical training on chaperone-dependent protein refolding

**FIGURE 6:**
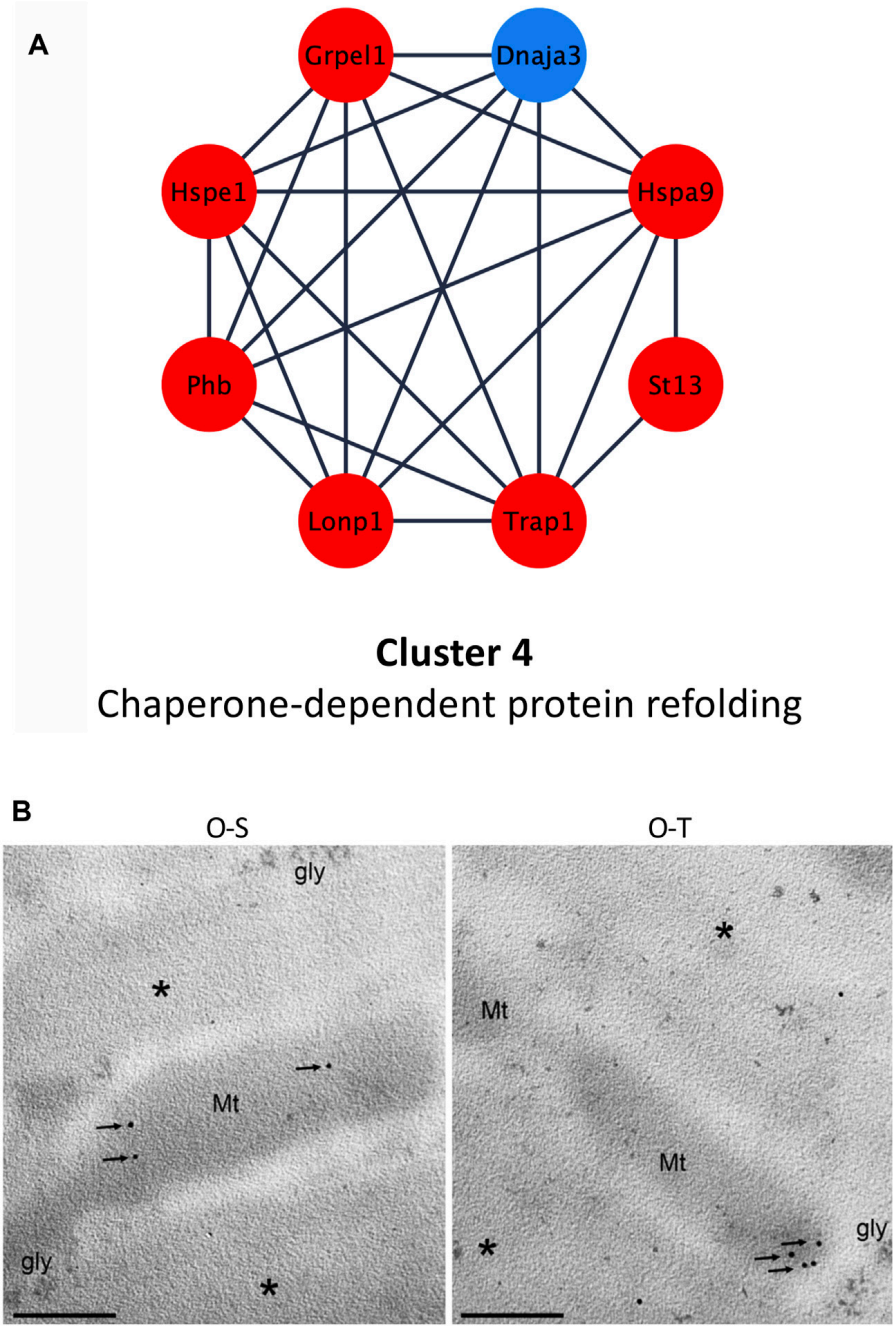
Chaperone-dependent protein refolding. **(A)** Differential expression of proteins belonging to cluster 4 showing up- (red) and down- (blue) regulated proteins in trained compared to sedentary mice. **(B)** mtHsp70 immunolabeling on aldehyde-fixed and LRWhite-embedded samples. Representative ultrastructural images of mtHsp70 immunolabeling (arrows) on mitochondria (Mt) in old sedentary (O-S) and old trained (O-T) mice. *myofibrils; gly, glycogen deposit. Bar: 200 nm.

Among chaperon proteins, heat shock proteins (HPSs), in stress conditions, are rapidly induced to stabilize misfolded or denatured polypeptides (Stetler et al., 2010), but also in normal conditions are involved, for instance, in folding and maturation of synthesized proteins. Hspe1 and Hspa9 were upregulated (log_2_Fold change O-T/O-S = 0.5995 and 0.7155, respectively) after training (Figure 6A; Supplementary Table S3).

It is known that during aging there is an increased oxidative stress which determines damages to biological macromolecules (*e.g.,* protein carbonylation). Kayani and collaborators, for instance, demonstrated that muscles of adult and old mice overexpressing Hspe1/Hsp10 showed a lower susceptibility to contraction-induced damages, because this molecule, providing a protection against ROS, reduces the mitochondrial protein carbonyl content (Kayani et al., 2010).

In agreement with these observations, the upregulation of Hspe1 and of several mitochondrial components (*e.g.,* proteins belonging to cluster 1), demonstrated in our experimental model, may contribute to preserve an efficient mitochondrial function in O-T.

In addition, adapted physical training determined an upregulation of Hspa9 (also named Grp75 or mortalin or mtHsp70). This chaperone is mainly localized into mitochondria, and its expression correlates with increased mitochondrial activity (Ornatsky et al., 1995). Moreover, it is involved in several functions such as: i) protein transfer from nucleus to mitochondria (Dolezal et al., 2006), ii) energy metabolism (Xu et al., 2009), iii) proteasomal degradation, iv) immune response (Pilzer and Fishelson, 2005) and v) life span extension (Kaula et al., 2000). It has been shown that mortalin is over-expressed under various stress conditions (*e.g.,* caloric restriction, low doses of ionizing radiation) (Ornatsky et al., 1995; Merrick et al., 1997; Sadekova et al., 1997) and also after physical exercise, suggesting that Hspa9/mortalin exerts a cytoprotective effect counteracting ROS accumulation (Liu et al., 2005; Xu et al., 2009). Moreover, the increased expression of Hspa9 favors mitochondrial protein biogenesis in synergy with the upregulation of co-chaperones (*e.g.,* Grpel) and of LONP1 (Wiedemann and Pfanner, 2017; Shin et al., 2021), a serine protease that mediates the selective degradation of misfolded/unfolded/oxidized proteins (De Gaetano et al., 2020; Gibellini et al., 2020), possibly reducing mitochondrial proteotoxicity. These data are in agreement with the upregulation of Grpel (log_2_Fold change O-T/O-S = 0.722) and Lonp1 (log_2_Fold change O-T/O-S = 1.1642) in trained mice (Figure 6A; Supplementary Table S3).

MtHsp70 immunolabelling per mitochondrion (Figure 6B) did not reveal significant differences between the two experimental groups (20.70 ± 2.88 vs*.* 16.95 ± 4.71 *p* = 0.5). This result seems to contradict the proteomic data (*i.e.,* upregulation of this proteins in O-T vs*.* O-S). This apparent discrepancy is because immunolabelling considers the protein quantity per mitochondrion, while MS-based quantitative proteomics data refer to the whole muscle in which the mitochondrial density is significantly increased in O-T vs*.* O-S (Figure 3C).

### 3.7 Effects of adapted physical training on collagen-activated signaling pathways

Although intramuscular connective tissue accounts for 1%–10% of skeletal muscle, the amount as well as orientation/organization of ECM is important to regulate the stiffness of the tissue and to transmit forces for optimal contraction (Kjaer, 2004).

**FIGURE 7:**
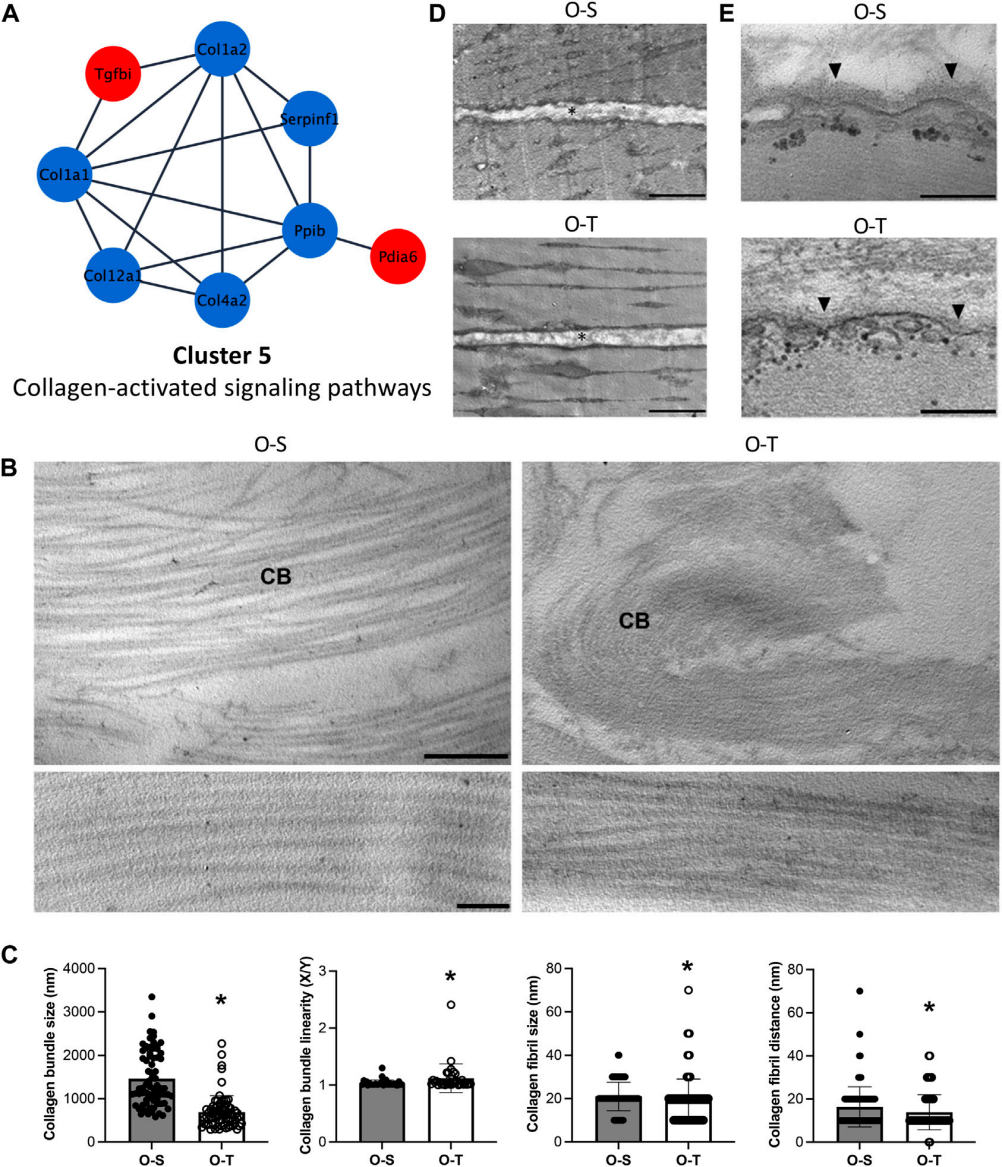
Collagen proteomics and ultrastructure. **(A)** Differential expression of proteins belonging to cluster 5 showing the up- (red) and down- (blue) regulated proteins in trained compared to sedentary mice. **(B)** Representative ultrastructural images of the perimysium of old sedentary (O-S) and old trained (O-T) GAST muscle. CB, collagen bundle. Bar: 1 µm (upper panels), bar: 100 nm (lower panels). **(C)** Morphometric analysis of collagen bundles and collagen fibrils. X/Y, ratio between the real length of the collagen bundle profile and the corresponding linear length. *p < 0.05. **(D)** Representative ultrastructural images of the endomysium (*) in old sedentary (O-S) and old trained (O-T) GAST muscle. Scatter dot plots show individual values, mean ± SD. **(E)** The basement membrane (arrowhead) covering the myofiber sarcolemma. Bars: 500 nm.

It is therefore intriguing to observe that adapted physical training in old mice significantly reduced some collagen types (*e.g.,* Col1a2, Col1a1, Col4a2, and Col12a1) (log_2_Fold change O-T/O-S = −0.8333, −0.7198, −0.9642, and −0.8735, respectively) (Figure 7A; Supplementary Table S3).

Collagen type I is the most represented collagen within skeletal muscles, whereas type IV is typically localized in the basement membranes adjacent to the sarcolemma. Interestingly, collagen type XII is highly expressed in muscles where it interacts with the surface of collagen type I fibrils regulating collagen fibril spacing and assembly during fibrillogenesis (Izu and Birk, 2023). The general reduction of all these collagens after exercise could be due to lower synthesis of these molecules and/or to increased susceptibility to proteolytic activities and turnover (*e.g.,* MMP and cathepsins) as already observed (Rullman et al., 2009; Guzzoni et al., 2018).

The ECM of the perimysium is organized in a network of collagen bundles (Figure 7B) that appeared not only significantly thinner (*p* < 0.001) and less linear (*p* = 0.004) in O-T vs*.* O-S, but also formed by collagen fibrils significantly smaller (*p* = 0.0018) and closer to each other (*p* = 0.003) in O-T vs. O-S (Figure 7C). These changes may reflect the lower presence of collagen type I and type XII after training. Therefore, the increase of ECM components, associated with the age-dependent enlargement of interfibrillar spacing (Lofaro et al., 2021), is counteracted/limited by adapted physical training in agreement with the improved turnover and assembly of ECM components (Rullman et al., 2009; Guzzoni et al., 2018). The association of skeletal muscle aging with significant decrease in collagen fiber “tortuosity” (Stearns-Reider et al., 2017) and accumulation of extensively cross-linked collagen as cause of increased muscle stiffness (Wood et al., 2014; Csapo et al., 2020) has been widely demonstrated. Therefore, the collagen bundle rearrangement in O-T mice, characterized by significantly thinner and more tortuous collagen bundles composed of smaller and more compact fibrils, may be a positive effect counteracting typical hallmarks of muscle aging (Wood et al., 2014).

In both O-S and O-T, the endomysium is a network of collagen fibrils connected to the basement membrane. Morphometrical analyses of the endomysium thickness, performed measuring the distance between two adjacent longitudinally sectioned myofibers, showed no significant difference in O-S vs*.* O-T (Figure 7D). The basement membrane, which covers the surface of each myofiber as an electrodense sheath, was significantly thinner in O-T vs. O-S (30.32 ± 0.58 nm vs*.* 41.88 ± 1.07 nm; *p* < 0.001) (Figure 7E). Within this context, the reduction of type IV collagen in O-T, observed by proteomic analysis and consistent with a thinner basement membrane found by ultrastructural morphometry, indicates that physical training positively influenced the ECM remodeling (Kjaer, 2004; Heinemeier et al., 2009; Hyldahl et al., 2015), as previously observed (Carmeli et al., 2005; Rullman et al., 2009; Ogasawara et al., 2014).

## 4 Conclusion

We are aware that this study shows some limitations: i) the results are related to GAST muscle without comparison with other muscle types (*e.g.,* soleus); ii) proteomic data and morphometric analyses do not provide functional information; iii) we considered male mice, and therefore we cannot exclude the possibility that the effect of exercise on female mice may have a different impact on GAST muscle; iv) data provide a static picture of protein concentration and/or distribution at one point-time and do not capture the dynamic process of changes (*i.e.,* how the protein increase or decrease over time); v) analyses did not focus on protein post-translational modifications which are known to affect protein behavior and characteristics (*e.g.,* protein lifespan, solubility, and assembly).

However, this study has many strengths related to: i) use of different extraction buffer to improve the isolation of both soluble and insoluble proteins; ii) a label free quantification that allows to directly compare the peptide intensities between the samples since data are obtained under identical conditions using high resolution MS/MS; iii) use bioinformatic software to identify the protein-protein interaction of DEPs and the process/pathways in which DEPs are involved; iv) morphological and morphometric analyses which allow to visualize the ultrastructural changes and the localization of proteins in the two experimental conditions (*i.e.,* O-S and O-T).

In summary, data indicate that the GAST muscle positively responds to adapted physical training started at old age, being capable: i) to enhance the size of myofibers and the expression of regulatory proteins supporting sarcolemmal integrity; ii) to increase the density of mitochondria and to modify the expression of mitochondrial components; iii) to reduce the expression of ribosomal proteins and to affect the nucleolar organization establishing protective mechanisms lowering the risk of accumulating damaged proteins; iv) to favor ECM remodeling. Therefore, this study provides important insights on the targets of adapted physical training, which can be regarded as suitable benchmarks for future *in vivo* studies further exploring the effects of this type of physical activity by functional/metabolic approaches.

Finally, this study reinforces that an adapted physical training initiated in old life can counteract some markers of the aged skeletal muscle, underlining the importance to adopt a physically active behavior at any age. In addition, future studies focused on microvascular characteristics and transcriptome-wide profiling (*e.g.,* mRNA, microRNA, and lncRNA) will allow to advance our current understanding of adapted physical training in old age.

## Data Availability

The data presented in the study are deposited in the ProteomeXchange (PRIDE) repository, accession number:PXD045483.

## References

[B1] Bassel-Duby R. Olson E. N. (2006). Signaling pathways in skeletal muscle remodeling. Annu. Rev. Biochem. 75, 19–37. 10.1146/annurev.biochem.75.103004.142622 16756483

[B2] Batsis J. A. Mackenzie T. A. Barre L. K. Lopez-Jimenez F. Bartels S. J. (2014). Sarcopenia, sarcopenic obesity and mortality in older adults: results from the National Health and Nutrition Examination Survey III. Eur. J. Clin. Nutr. 68, 1001–1007. 10.1038/ejcn.2014.117 24961545

[B3] Beaudart C. Dawson A. Shaw S. C. Harvey N. C. Kanis J. A. Binkley N. (2017). Nutrition and physical activity in the prevention and treatment of sarcopenia: systematic review. Osteoporos. Int. 28, 1817–1833. 10.1007/s00198-017-3980-9 28251287 PMC5457808

[B4] Boraldi F. Lofaro F. D. Accorsi A. Ross E. Malagoli D. (2019). Toward the molecular deciphering of Pomacea canaliculata immunity: first proteomic analysis of circulating hemocytes. Proteomics 19, e1800314. 10.1002/pmic.201800314 30537342

[B5] Bradford M. M. (1976). A rapid and sensitive method for the quantitation of microgram quantities of protein utilizing the principle of protein-dye binding. Anal. Biochem. 72, 248–254. 10.1006/abio.1976.9999 942051

[B6] Briguet A. Courdier-Fruh I. Foster M. Meier T. Magyar J. P. (2004). Histological parameters for the quantitative assessment of muscular dystrophy in the mdx-mouse. Neuromuscul. Disord. 14, 675–682. 10.1016/j.nmd.2004.06.008 15351425

[B7] Buffa P. Guarriera-Bobyleva V. Muscatello U. Pasquali-Ronchetti I. (1970). Conformational changes of mitochondria associated with uncoupling of oxidative phosphorylation *in vivo* and *in vitro* . Nature 226, 272–274. 10.1038/226272a0 4191173

[B8] Carmeli E. Moas M. Lennon S. Powers S. K. (2005). High intensity exercise increases expression of matrix metalloproteinases in fast skeletal muscle fibres. Exp. Physiol. 90, 613–619. 10.1113/expphysiol.2004.029462 15833756

[B9] Chen L.-K. Woo J. Assantachai P. Auyeung T.-W. Chou M.-Y. Iijima K. (2020). Asian working group for sarcopenia: 2019 consensus update on sarcopenia diagnosis and treatment. J. Am. Med. Dir. Assoc. 21, 300–307. 10.1016/j.jamda.2019.12.012 32033882

[B10] Cruz-Jentoft A. J. Bahat G. Bauer J. Boirie Y. Bruyère O. Cederholm T. (2019). Sarcopenia: revised European consensus on definition and diagnosis. Age Ageing 48, 16–31. 10.1093/ageing/afy169 30312372 PMC6322506

[B11] Cruz-Jentoft A. J. Sayer A. A. (2019). Sarcopenia. Lancet 393, 2636–2646. 10.1016/S0140-6736(19)31138-9 31171417

[B12] Csapo R. Gumpenberger M. Wessner B. (2020). Skeletal muscle extracellular matrix - what do we know about its composition, regulation, and physiological roles? A narrative review. Front. Physiol. 11, 253. 10.3389/fphys.2020.00253 32265741 PMC7096581

[B13] De Gaetano A. Gibellini L. Bianchini E. Borella R. De Biasi S. Nasi M. (2020). Impaired mitochondrial morphology and functionality in Lonp1wt/- mice. J. Clin. Med. 9, 1783. 10.3390/jcm9061783 32521756 PMC7355737

[B14] Distefano G. Standley R. A. Zhang X. Carnero E. A. Yi F. Cornnell H. H. (2018). Physical activity unveils the relationship between mitochondrial energetics, muscle quality, and physical function in older adults. J. Cachexia Sarcopenia Muscle 9, 279–294. 10.1002/jcsm.12272 29368427 PMC5879963

[B15] Dolezal P. Likic V. Tachezy J. Lithgow T. (2006). Evolution of the molecular machines for protein import into mitochondria. Science 313, 314–318. 10.1126/science.1127895 16857931

[B16] Doran P. Donoghue P. O’Connell K. Gannon J. Ohlendieck K. (2009). Proteomics of skeletal muscle aging. Proteomics 9, 989–1003. 10.1002/pmic.200800365 19180535

[B17] Egan B. Zierath J. R. (2013). Exercise metabolism and the molecular regulation of skeletal muscle adaptation. Cell Metab. 17, 162–184. 10.1016/j.cmet.2012.12.012 23395166

[B18] FabeneP. F.MariottiR.Navarro MoraG.ChakirA.ZancanaroC. (2008). Forced mild physical training-induced effects on cognitive and locomotory behavior in old mice. J. Nutr. Health Aging 12, 388–390. 10.1007/BF02982671 18548176

[B19] Feng S. Zhou L. Huang C. Xie K. Nice E. C. (2015). Interactomics: toward protein function and regulation. Expert Rev. Proteomics 12, 37–60. 10.1586/14789450.2015.1000870 25578092

[B20] Figueiredo V. C. Wen Y. Alkner B. Fernandez-Gonzalo R. Norrbom J. Vechetti I. J. (2021). Genetic and epigenetic regulation of skeletal muscle ribosome biogenesis with exercise. J. Physiol. 599, 3363–3384. 10.1113/JP281244 33913170

[B21] Galkin A. Dröse S. Brandt U. (2006). The proton pumping stoichiometry of purified mitochondrial complex I reconstituted into proteoliposomes. Biochim. Biophys. Acta 1757, 1575–1581. 10.1016/j.bbabio.2006.10.001 17094937

[B22] Gao H.-E. Wu D.-S. Sun L. Yang L.-D. Qiao Y. Ma S. (2020). Effects of lifelong exercise on age-related body composition, oxidative stress, inflammatory cytokines, and skeletal muscle proteome in rats. Mech. Ageing Dev. 189, 111262. 10.1016/j.mad.2020.111262 32422206

[B23] Gibala M. J. McGee S. L. Garnham A. P. Howlett K. F. Snow R. J. Hargreaves M. (2009). Brief intense interval exercise activates AMPK and p38 MAPK signaling and increases the expression of PGC-1alpha in human skeletal muscle. J. Appl. Physiol. (1985) 106, 929–934. 10.1152/japplphysiol.90880.2008 19112161

[B24] Gibellini L. De Gaetano A. Mandrioli M. Van Tongeren E. Bortolotti C. A. Cossarizza A. (2020). The biology of Lonp1: more than a mitochondrial protease. Int. Rev. Cell Mol. Biol. 354, 1–61. 10.1016/bs.ircmb.2020.02.005 32475470

[B25] Glancy B. Balaban R. S. (2021). Energy metabolism design of the striated muscle cell. Physiol. Rev. 101, 1561–1607. 10.1152/physrev.00040.2020 33733879 PMC8576364

[B26] Glancy B. Hartnell L. M. Malide D. Yu Z.-X. Combs C. A. Connelly P. S. (2015). Mitochondrial reticulum for cellular energy distribution in muscle. Nature 523, 617–620. 10.1038/nature14614 26223627 PMC6988728

[B27] Glancy B. Kim Y. Katti P. Willingham T. B. (2020). The functional impact of mitochondrial structure across subcellular scales. Front. Physiology 11, 541040. 10.3389/fphys.2020.541040 PMC7686514 33262702

[B28] Goessens G. (1984). Nucleolar structure. Int. Rev. Cytol. 87, 107–158. 10.1016/s0074-7696(08)62441-9 6201455

[B29] Gonskikh Y. Polacek N. (2017). Alterations of the translation apparatus during aging and stress response. Mech. Ageing Dev. 168, 30–36. 10.1016/j.mad.2017.04.003 28414025

[B30] Gonzalez-Freire M. Semba R. D. Ubaida-Mohien C. Fabbri E. Scalzo P. Højlund K. (2017). The human skeletal muscle proteome project: a reappraisal of the current literature. J. Cachexia Sarcopenia Muscle 8, 5–18. 10.1002/jcsm.12121 27897395 PMC5326819

[B31] Graziano F. Juhasz V. Brunetti G. Cipriani A. Szabo L. Merkely B. (2022)). Strenuous endurance sports activity damage the cardiovascular system of healthy athletes? A narrative review. J. Cardiovasc Dev. Dis. 9, 347. 10.3390/jcdd9100347 36286299 PMC9604467

[B32] Gueugneau M. Coudy-Gandilhon C. Gourbeyre O. Chambon C. Combaret L. Polge C. (2014). Proteomics of muscle chronological ageing in post-menopausal women. BMC Genomics 15, 1165. 10.1186/1471-2164-15-1165 25532418 PMC4523020

[B33] Guzzoni V. Ribeiro M. B. T. Lopes G. N. de Cássia Marqueti R. de Andrade R. V. Selistre-de-Araujo H. S. (2018). Effect of resistance training on extracellular matrix adaptations in skeletal muscle of older rats. Front. Physiology 9, 374. 10.3389/fphys.2018.00374 PMC5904267 29695977

[B34] Hansson B. Olsen L. A. Nicoll J. X. von Walden F. Melin M. Strömberg A. (2019). Skeletal muscle signaling responses to resistance exercise of the elbow extensors are not compromised by a preceding bout of aerobic exercise. Am. J. Physiol. Regul. Integr. Comp. Physiol. 317, R83-R92. 10.1152/ajpregu.00022.2019 30969843

[B35] Heinemeier K. M. Olesen J. L. Haddad F. Schjerling P. Baldwin K. M. Kjaer M. (2009). Effect of unloading followed by reloading on expression of collagen and related growth factors in rat tendon and muscle. J. Appl. Physiol. (1985) 106, 178–186. 10.1152/japplphysiol.91092.2008 18988763

[B36] Hipkiss A. R. (2007). On why decreasing protein synthesis can increase lifespan. Mech. Ageing Dev. 128, 412–414. 10.1016/j.mad.2007.03.002 17452047

[B37] Holloway K. V. O’Gorman M. Woods P. Morton J. P. Evans L. Cable N. T. (2009). Proteomic investigation of changes in human vastus lateralis muscle in response to interval-exercise training. Proteomics 9, 5155–5174. 10.1002/pmic.200900068 19834892

[B38] Hyldahl R. D. Nelson B. Xin L. Welling T. Groscost L. Hubal M. J. (2015). Extracellular matrix remodeling and its contribution to protective adaptation following lengthening contractions in human muscle. FASEB J. 29, 2894–2904. 10.1096/fj.14-266668 25808538

[B39] Imagita H. Nishii Y. Fujita N. Sukedzane T. Kawata S. (2020). Effects of appropriate-intensity treadmill exercise on skeletal muscle and respiratory functions in a rat model of emphysema. Biomed. Res. 41, 13–22. 10.2220/biomedres.41.13 32092736

[B40] IzquierdoM.MerchantR. A.MorleyJ. E.AnkerS. D.AprahamianI.AraiH. (2021). International exercise recommendations in older adults (ICFSR): expert consensus guidelines. J. Nutr. Health Aging 25, 824–853. 10.1007/s12603-021-1665-8 34409961

[B41] Izu Y. Birk D. E. (2023). Collagen XII mediated cellular and extracellular mechanisms in development, regeneration, and disease. Front. Cell Dev. Biol. 11, 1129000. 10.3389/fcell.2023.1129000 36936682 PMC10017729

[B42] Jamison J. M. Gilloteaux J. Perlaky L. Thiry M. Smetana K. Neal D. (2010). Nucleolar changes and fibrillarin redistribution following apatone treatment of human bladder carcinoma cells. J. Histochem Cytochem 58, 635–651. 10.1369/jhc.2010.956284 20385787 PMC2889405

[B43] Kaula S. C. Reddelb R. R. Sugiharac T. Mitsuia Y. Wadhwac R. (2000). Inactivation of p53 and life span extension of human diploid fibroblasts by mot-2. FEBS Lett. 474, 159–164. 10.1016/s0014-5793(00)01594-5 10838077

[B44] Kayani A. C. Close G. L. Dillmann W. H. Mestril R. Jackson M. J. McArdle A. (2010). Overexpression of HSP10 in skeletal muscle of transgenic mice prevents the age-related fall in maximum tetanic force generation and muscle cross-sectional area. Am. J. Physiol. Regul. Integr. Comp. Physiol. 299, R268–R276. 10.1152/ajpregu.00334.2009 20410481 PMC2904147

[B45] Kjaer M. (2004). Role of extracellular matrix in adaptation of tendon and skeletal muscle to mechanical loading. Physiol. Rev. 84, 649–698. 10.1152/physrev.00031.2003 15044685

[B46] Kleinert M. Parker B. L. Jensen T. E. Raun S. H. Pham P. Han X. (2018). Quantitative proteomic characterization of cellular pathways associated with altered insulin sensitivity in skeletal muscle following high-fat diet feeding and exercise training. Sci. Rep. 8, 10723. 10.1038/s41598-018-28540-5 30013070 PMC6048112

[B47] Lacavalla M. A. Cisterna B. (2023). Uranyl-free staining as a suitable contrasting technique for nuclear structures at transmission electron microscopy. Methods Mol. Biol. 2566, 225–231. 10.1007/978-1-0716-2675-7_18 36152255

[B48] La Gerche A. Rakhit D. J. Claessen G. (2017). Exercise and the right ventricle: a potential Achilles’ heel. Cardiovasc Res. 113, 1499–1508. 10.1093/cvr/cvx156 28957535

[B49] Landi F. Cruz-Jentoft A. J. Liperoti R. Russo A. Giovannini S. Tosato M. (2013). Sarcopenia and mortality risk in frail older persons aged 80 years and older: results from ilSIRENTE study. Age Ageing 42, 203–209. 10.1093/ageing/afs194 23321202

[B50] Lane N. Martin W. (2010). The energetics of genome complexity. Nature 467, 929–934. 10.1038/nature09486 20962839

[B51] Lang F. Aravamudhan S. Nolte H. Türk C. Hölper S. Müller S. (2017). Dynamic changes in the mouse skeletal muscle proteome during denervation-induced atrophy. Dis. Model Mech. 10, 881–896. 10.1242/dmm.028910 28546288 PMC5536905

[B52] Lanza I. R. Short D. K. Short K. R. Raghavakaimal S. Basu R. Joyner M. J. (2008). Endurance exercise as a countermeasure for aging. Diabetes 57, 2933–2942. 10.2337/db08-0349 18716044 PMC2570389

[B53] Lee S. M. Lee M. C. Bae W. R. Yoon K. J. Moon H. Y. (2022). Muscle fiber type-dependence effect of exercise on genomic networks in aged mice models. Aging (Albany NY) 14, 3337–3364. 10.18632/aging.204024 35440516 PMC9085230

[B54] Levett D. Z. H. Viganò A. Capitanio D. Vasso M. De Palma S. Moriggi M. (2015). Changes in muscle proteomics in the course of the caudwell research expedition to Mt. Everest. Proteomics 15, 160–171. 10.1002/pmic.201400306 25370915

[B55] Lexell J. (1995). Human aging, muscle mass, and fiber type composition. J. Gerontol. A Biol. Sci. Med. Sci. 50, 11–16. 10.1093/gerona/50a.special_issue.11 7493202

[B56] Li F.-H. Sun L. Wu D.-S. Gao H.-E. Min Z. (2019). Proteomics-based identification of different training adaptations of aged skeletal muscle following long-term high-intensity interval and moderate-intensity continuous training in aged rats. Aging (Albany NY) 11, 4159–4182. 10.18632/aging.102044 31241467 PMC11623340

[B57] Liu Y. Liu W. Song X.-D. Zuo J. (2005). Effect of GRP75/mthsp70/PBP74/mortalin overexpression on intracellular ATP level, mitochondrial membrane potential and ROS accumulation following glucose deprivation in PC12 cells. Mol. Cell Biochem. 268, 45–51. 10.1007/s11010-005-2996-1 15724436

[B58] Lofaro F. D. Boraldi F. Garcia-Fernandez M. Estrella L. Valdivielso P. Quaglino D. (2020). Relationship between mitochondrial structure and bioenergetics in pseudoxanthoma elasticum dermal fibroblasts. Front. Cell Dev. Biol. 8, 610266. 10.3389/fcell.2020.610266 33392199 PMC7773789

[B59] Lofaro F. D. Cisterna B. Lacavalla M. A. Boschi F. Malatesta M. Quaglino D. (2021). Age-related changes in the matrisome of the mouse skeletal muscle. Int. J. Mol. Sci. 22, 10564. 10.3390/ijms221910564 34638903 PMC8508832

[B60] López-Otín C. Blasco M. A. Partridge L. Serrano M. Kroemer G. (2013). The hallmarks of aging. Cell 153, 1194–1217. 10.1016/j.cell.2013.05.039 23746838 PMC3836174

[B61] Massett M. P. Matejka C. Kim H. (2021). Systematic review and meta-analysis of endurance exercise training protocols for mice. Front. Physiol. 12, 782695. 10.3389/fphys.2021.782695 34950054 PMC8691460

[B62] Merrick B. A. Walker V. R. He C. Patterson R. M. Selkirk J. K. (1997). Induction of novel Grp75 isoforms by 2-deoxyglucose in human and murine fibroblasts. Cancer Lett. 119, 185–190. 10.1016/s0304-3835(97)00270-x 9570370

[B63] Mesquita P. H. C. Vann C. G. Phillips S. M. McKendry J. Young K. C. Kavazis A. N. (2021). Skeletal muscle ribosome and mitochondrial biogenesis in response to different exercise training modalities. Front. Physiology 12, 725866. 10.3389/fphys.2021.725866 PMC8504538 34646153

[B64] Mikkelsen U. R. Couppé C. Karlsen A. Grosset J. F. Schjerling P. Mackey A. L. (2013). Life-long endurance exercise in humans: circulating levels of inflammatory markers and leg muscle size. Mech. Ageing Dev. 134, 531–540. 10.1016/j.mad.2013.11.004 24287006

[B65] Morrison P. R. Biggs R. B. Booth F. W. (1989). Daily running for 2 wk and mRNAs for cytochrome c and alpha-actin in rat skeletal muscle. Am. J. Physiol. 257, C936–C939. 10.1152/ajpcell.1989.257.5.C936 2480716

[B66] Müller W. (1976). Subsarcolemmal mitochondria and capillarization of soleus muscle fibers in young rats subjected to an endurance training. A morphometric study of semithin sections. Cell Tissue Res. 174, 367–389. 10.1007/BF00220682 1000581

[B67] Murgia M. Toniolo L. Nagaraj N. Ciciliot S. Vindigni V. Schiaffino S. (2017). Single muscle fiber proteomics reveals fiber-type-specific features of human muscle aging. Cell Rep. 19, 2396–2409. 10.1016/j.celrep.2017.05.054 28614723

[B68] Muscatello U. Pasquali-Ronchetti I. (1972). The relation between structure and function in mitochondria. Its relevance in pathology. Pathobiol. Annu. 2, 1–46. 4359245

[B69] Nielsen J. Gejl K. D. Hey-Mogensen M. Holmberg H.-C. Suetta C. Krustrup P. (2017). Plasticity in mitochondrial cristae density allows metabolic capacity modulation in human skeletal muscle. J. Physiol. 595, 2839–2847. 10.1113/JP273040 27696420 PMC5407961

[B70] Novelli M. Pocai A. Skalicky M. Viidik A. Bergamini E. Masiello P. (2004). Effects of life-long exercise on circulating free fatty acids and muscle triglyceride content in ageing rats. Exp. Gerontol. 39, 1333–1340. 10.1016/j.exger.2004.06.014 15489056

[B71] Ogasawara R. Nakazato K. Sato K. Boppart M. D. Fujita S. (2014). Resistance exercise increases active MMP and β1-integrin protein expression in skeletal muscle. Physiol. Rep. 2, e12212. 10.14814/phy2.12212 25413329 PMC4255818

[B72] Ohlendieck K. (2011). Proteomic profiling of fast-to-slow muscle transitions during aging. Front. Physiology 2, 105. 10.3389/fphys.2011.00105 PMC3245893 22207852

[B73] Ornatsky O. I. Connor M. K. Hood D. A. (1995). Expression of stress proteins and mitochondrial chaperonins in chronically stimulated skeletal muscle. Biochem. J. 311 (1), 119–123. 10.1042/bj3110119 7575442 PMC1136127

[B74] Petriz B. A. Gomes C. P. C. Almeida J. A. de Oliveira G. P. Ribeiro F. M. Pereira R. W. (2017). The effects of acute and chronic exercise on skeletal muscle proteome. J. Cell Physiol. 232, 257–269. 10.1002/jcp.25477 27381298

[B75] Pilzer D. Fishelson Z. (2005). Mortalin/GRP75 promotes release of membrane vesicles from immune attacked cells and protection from complement-mediated lysis. Int. Immunol. 17, 1239–1248. 10.1093/intimm/dxh300 16091382

[B76] Proctor D. N. Sinning W. E. Walro J. M. Sieck G. C. Lemon P. W. (1995). Oxidative capacity of human muscle fiber types: effects of age and training status. J. Appl. Physiol. (1985) 78, 2033–2038. 10.1152/jappl.1995.78.6.2033 7665396

[B77] Rasmussen U. F. Krustrup P. Kjaer M. Rasmussen H. N. (2003). Experimental evidence against the mitochondrial theory of aging. A study of isolated human skeletal muscle mitochondria. Exp. Gerontol. 38, 877–886. 10.1016/s0531-5565(03)00092-5 12915209

[B78] Recher L. Whitescarver J. Briggs L. (1969). The fine structure of a nucleolar constituent. J. Ultrastruct. Res. 29, 1–14. 10.1016/s0022-5320(69)80052-3 4900225

[B79] Robinson S. M. Reginster J. Y. Rizzoli R. Shaw S. C. Kanis J. A. Bautmans I. (2018). Does nutrition play a role in the prevention and management of sarcopenia? Clin. Nutr. 37, 1121–1132. 10.1016/j.clnu.2017.08.016 28927897 PMC5796643

[B80] Rullman E. Norrbom J. Strömberg A. Wågsäter D. Rundqvist H. Haas T. (2009). Endurance exercise activates matrix metalloproteinases in human skeletal muscle. J. Appl. Physiol. (1985) 106, 804–812. 10.1152/japplphysiol.90872.2008 19131480

[B81] Russ D. W. Kent-Braun J. A. (2004). Is skeletal muscle oxidative capacity decreased in old age? Sports Med. 34, 221–229. 10.2165/00007256-200434040-00002 15049714

[B82] Sadekova S. Lehnert S. Chow T. Y. (1997). Induction of PBP74/mortalin/Grp75, a member of the hsp70 family, by low doses of ionizing radiation: a possible role in induced radioresistance. Int. J. Radiat. Biol. 72, 653–660. 10.1080/095530097142807 9416787

[B83] Safdar A. Hamadeh M. J. Kaczor J. J. Raha S. Debeer J. Tarnopolsky M. A. (2010). Aberrant mitochondrial homeostasis in the skeletal muscle of sedentary older adults. PLoS One 5, e10778. 10.1371/journal.pone.0010778 20520725 PMC2875392

[B84] Schild M. Ruhs A. Beiter T. Zügel M. Hudemann J. Reimer A. (2015). Basal and exercise induced label-free quantitative protein profiling of m. vastus lateralis in trained and untrained individuals. J. Proteomics 122, 119–132. 10.1016/j.jprot.2015.03.028 25857276

[B85] Schwarzacher H. G. Wachtler F. (1991). The functional significance of nucleolar structures. Ann. Genet. 34, 151–160. 1809221

[B86] Shin C.-S. Meng S. Garbis S. D. Moradian A. Taylor R. W. Sweredoski M. J. (2021). LONP1 and mtHSP70 cooperate to promote mitochondrial protein folding. Nat. Commun. 12, 265. 10.1038/s41467-020-20597-z 33431889 PMC7801493

[B87] Singhto N. Thongboonkerd V. (2018). Exosomes derived from calcium oxalate-exposed macrophages enhance IL-8 production from renal cells, neutrophil migration and crystal invasion through extracellular matrix. J. Proteomics 185, 64–76. 10.1016/j.jprot.2018.06.015 29953960

[B88] Son H. Kim H. Kim C. (2011). The effect of resistance and endurance training on muscle proteome expression in human skeletal muscle. Med. Sci. Sports Exerc. 43, 303. 10.1249/01.MSS.0000400833.85126.de 20581711

[B89] Starnes J. W. Parry T. L. O’Neal S. K. Bain J. R. Muehlbauer M. J. Honcoop A. (2017). Exercise-induced alterations in skeletal muscle, Heart, liver, and serum metabolome identified by non-targeted metabolomics analysis. Metabolites 7, E40. 10.3390/metabo7030040 PMC5618325 28786928

[B90] Stearns-Reider K. M. D’Amore A. Beezhold K. Rothrauff B. Cavalli L. Wagner W. R. (2017). Aging of the skeletal muscle extracellular matrix drives a stem cell fibrogenic conversion. Aging Cell 16, 518–528. 10.1111/acel.12578 28371268 PMC5418187

[B91] Stetler R. A. Gan Y. Zhang W. Liou A. K. Gao Y. Cao G. (2010). Heat shock proteins: cellular and molecular mechanisms in the central nervous system. Prog. Neurobiol. 92, 184–211. 10.1016/j.pneurobio.2010.05.002 20685377 PMC2939168

[B92] Subramaniam J. Yamankurt G. Cunha S. R. (2022). Obscurin regulates ankyrin macromolecular complex formation. J. Mol. Cell Cardiol. 168, 44–57. 10.1016/j.yjmcc.2022.04.008 35447147 PMC11057898

[B93] Szklarczyk D. Gable A. L. Nastou K. C. Lyon D. Kirsch R. Pyysalo S. (2021). The STRING database in 2021: customizable protein-protein networks, and functional characterization of user-uploaded gene/measurement sets. Nucleic Acids Res. 49, D605–D612. 10.1093/nar/gkaa1074 33237311 PMC7779004

[B94] Talbot J. Maves L. (2016). Skeletal muscle fiber type: using insights from muscle developmental biology to dissect targets for susceptibility and resistance to muscle disease. Wiley Interdiscip. Rev. Dev. Biol. 5, 518–534. 10.1002/wdev.230 27199166 PMC5180455

[B95] Tavernarakis N. (2007). Protein synthesis and aging: eIF4E and the soma vs. germline distinction. Cell Cycle 6, 1168–1171. 10.4161/cc.6.10.4230 17495543

[B96] Tavernarakis N. (2008). Ageing and the regulation of protein synthesis: a balancing act? Trends Cell Biol. 18, 228–235. 10.1016/j.tcb.2008.02.004 18346894

[B97] Tsai T.-H. Choi M. Banfai B. Liu Y. MacLean B. X. Dunkley T. (2020). Selection of features with consistent profiles improves relative protein quantification in mass spectrometry experiments. Mol. Cell Proteomics 19, 944–959. 10.1074/mcp.RA119.001792 32234965 PMC7261813

[B98] Tsekrekou M. Stratigi K. Chatzinikolaou G. (2017). The nucleolus: in genome maintenance and repair. Int. J. Mol. Sci. 18, 1411. 10.3390/ijms18071411 28671574 PMC5535903

[B99] Vissing K. Schjerling P. (2014). Simplified data access on human skeletal muscle transcriptome responses to differentiated exercise. Sci. Data 1, 140041. 10.1038/sdata.2014.41 25984345 PMC4432635

[B100] Wiedemann N. Pfanner N. (2017). Mitochondrial machineries for protein import and assembly. Annu. Rev. Biochem. 86, 685–714. 10.1146/annurev-biochem-060815-014352 28301740

[B101] Wilkinson S. B. Phillips S. M. Atherton P. J. Patel R. Yarasheski K. E. Tarnopolsky M. A. (2008). Differential effects of resistance and endurance exercise in the fed state on signalling molecule phosphorylation and protein synthesis in human muscle. J. Physiol. 586, 3701–3717. 10.1113/jphysiol.2008.153916 18556367 PMC2538832

[B102] Willingham T. B. Ajayi P. T. Glancy B. (2021). Subcellular specialization of mitochondrial form and function in skeletal muscle cells. Front. Cell Dev. Biol. 9, 757305. 10.3389/fcell.2021.757305 34722542 PMC8554132

[B103] Wood L. K. Kayupov E. Gumucio J. P. Mendias C. L. Claflin D. R. Brooks S. V. (2014). Intrinsic stiffness of extracellular matrix increases with age in skeletal muscles of mice. J. Appl. Physiol. (1985) 117, 363–369. 10.1152/japplphysiol.00256.2014 24994884 PMC4137235

[B104] Xu L. Voloboueva L. A. Ouyang Y. Emery J. F. Giffard R. G. (2009). Overexpression of mitochondrial Hsp70/Hsp75 in rat brain protects mitochondria, reduces oxidative stress, and protects from focal ischemia. J. Cereb. Blood Flow. Metab. 29, 365–374. 10.1038/jcbfm.2008.125 18985056 PMC3676940

[B105] Zampieri S. Pietrangelo L. Loefler S. Fruhmann H. Vogelauer M. Burggraf S. (2015). Lifelong physical exercise delays age-associated skeletal muscle decline. J. Gerontol. A Biol. Sci. Med. Sci. 70, 163–173. 10.1093/gerona/glu006 24550352

[B106] Zancanaro C. Mariotti R. Perdoni F. Nicolato E. Malatesta M. (2007). Physical training is associated with changes in nuclear magnetic resonance and morphometrical parameters of the skeletal muscle in senescent mice. Eur. J. Histochem 51, 305–310. 10.4081/1156 18162461

