# Supplementary figures and images for: Aged gastrocnemius muscle of mice positively responds to a late onset adapted physical training
Source: Front Cell Dev Biol. 2023 Nov 13;11:1273309. doi: 10.3389/fcell.2023.1273309 (PMC10679468; doi:10.3389/fcell.2023.1273309)

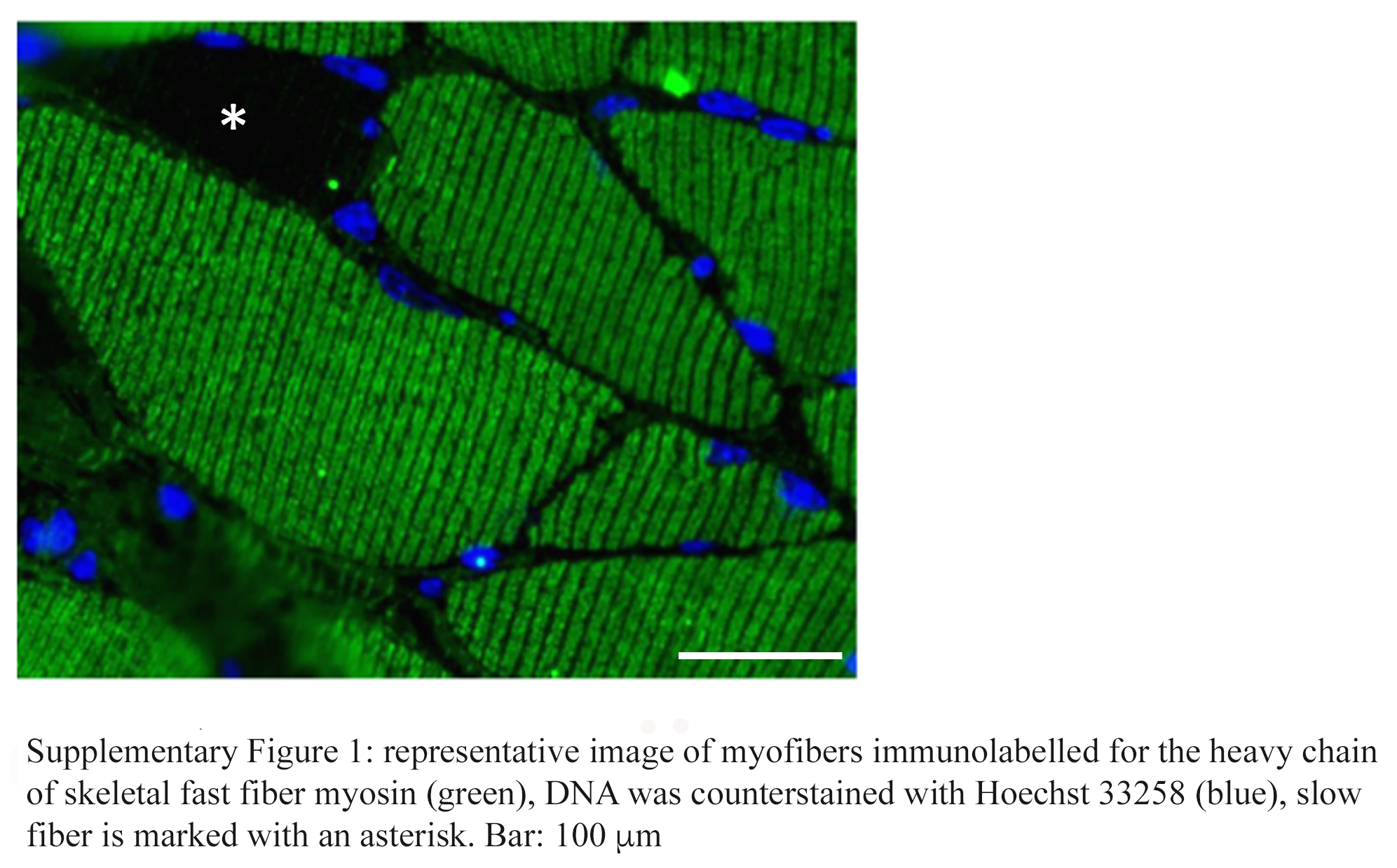

Supplement: Supplementary file 3 [file Image1.TIF]
